# The early transcriptome response of cassava (*Manihot esculenta* Crantz) to mealybug (*Phenacoccus manihoti*) feeding

**DOI:** 10.1371/journal.pone.0202541

**Published:** 2018-08-22

**Authors:** Molemi E. Rauwane, Damaris A. Odeny, Ian Millar, Chrissie Rey, Jasper Rees

**Affiliations:** 1 Biotechnology Platform, Agricultural Research Council, Onderstepoort, South Africa; 2 University of the Witwatersrand, Johannesburg, South Africa; 3 ICRISAT–Nairobi, Nairobi, Kenya; 4 Biosystematics Division, Plant Protection Research Institute, Agricultural Research Council, Queenswood, Pretoria, South Africa; Hochschule Geisenheim University, GERMANY

## Abstract

The mealybug, *Phenacoccus manihoti*, is a leading pest of cassava (*Manihot esculenta* Crantz), damaging this crop globally. Although the biological control of this mealybug using natural predators has been established, resistance breeding remains an important means of control. Understanding plant responses to insect herbivory, by determining and identifying differentially expressed genes (DEGs), is a vital step towards the understanding of molecular mechanisms of defence responses in plants and the development of resistant cultivars by gene editing. Morphological and molecular analysis confirmed the mealybug identity as *Phenacoccus manihoti* (Matile-Ferrero). The transcriptome response of the green mite resistant cassava genotype AR23.1 was compared to P40/1 with no known resistance at 24 and 72 hours of mealybug infestation compared to non-infested mock. A total of 301 and 206 genes were differentially expressed at 24 and 72 of mealybug infestation for AR23.1 and P40/1 genotypes respectively, using a log2 fold change and P-value ≤ 0.05. Gene ontology functional classification revealed an enrichment of genes in the secondary metabolic process category in AR23.1 in comparison with P40/1, while genes in the regulation of molecular function, cellular component biogenesis and electron carrier categories were more significantly enriched in P40/1 than in AR23.1. Biological pathway analysis, based on KEGG, revealed a significant enrichment of plant-pathogen interaction and plant hormonal signal transduction pathways for a cohort of up-regulated and down-regulated DEGs in both genotypes. Defence-related genes such as 2-oxogluterate, gibberellin oxidase and terpene synthase proteins were only induced in genotype AR23.1 and not in P40/1, and subsequently validated by RT-qPCR. The study revealed a difference in response to mealybug infestation in the two genotypes studied, with AR23.1 showing a higher number of differentially expressed transcripts post mealybug infestation at 24 and 72 hours. Candidate defence-related genes that were overexpressed in the AR23.1 genotype post mealybug infestation will be useful in future functional studies towards the control of mealybugs.

## Introduction

Cassava, tapioca or manioc (*Manihot esculenta* Crantz) is a woody perennial plant that feeds over 500 million people living throughout the tropics [1–4]. It is mainly cultivated for its large starchy roots but the leaves are also consumed as vegetable, especially in Africa. Although native to South America, cassava is a widely cultivated tuber crop in sub-Saharan Africa and an important food staple in terms of per capita food energy consumed [5, 6]. In South Africa, cassava is grown in Limpopo, Mpumalanga and KwaZulu-Natal provinces as a secondary crop. In the neighbouring South African countries such as Swaziland and Mozambique, cassava is a staple food crop and often used for industrial starch production. Cassava is grown by the resource-poor farmers in the seasonally humid regions of South Africa as a supplementary crop to maize (*Zea mays*).

Important abiotic stresses known to affect cassava production and quality include poor soils, post-harvest physiological deterioration [7], land degradation, low winter temperatures, low and variable annual rainfall. Biotic constraints include weeds, pests and pathogens [8–12]. Apart from the well-known viral pathogens causing cassava mosaic disease (CMD) and cassava brown streak disease (CBSD) [10], pests such as cassava green mite (*Mononychellus tanajoa*), whiteflies (*Bemisia tabaci* and *Aleurotrachelus socialis*), the root mealybug (*Planococcus citri*), and two species of cassava mealybug (*Phenacoccus manihoti* and *Phenacoccus herreni*) also reduce cassava yields [13, 14]. Due to these abiotic and biotic stresses, the production of cassava as a food security crop in sub-Saharan Africa and globally is currently at risk.

*Phenacoccus manihoti* (*P*. *manihoti)* have been reported to occur in South Africa [15, 16] and its presence further validated using PCR [15, 16]. *P*. *manihoti* was accidentally introduced into Africa in the 1970s, and became a major pest in most cassava growing regions [17] causing yield losses as high as 80%. The pest causes cassava yield reductions [18, 19] by injecting toxic saliva into the plant during feeding [20]. Initially, the control of mealybugs was undertaken using insecticides. Biological control was later introduced using the parasitoid wasp *Apoanagyrus lopezi* with some success [18]. Regardless of the success in biological control of CMB, growing resistant cultivars remains the most sustainable option for managing the pests.

Plant defence response to herbivorous insects is generally activated at the site of insect infestation or herbivore feeding. Following contact with the plant’s surface, the insect can release and inject biological components (or other elicitors) which can trigger plant immune responses such as jasmonic acid (JA), ethylene (ET) and salicylic acid (SA) pathways, which are the major signal cascades involved in plant defence against pathogens and pests [21–24]. The cross talk between the signal pathways results in the release of volatiles that further activate the expression of defence-related genes. Production of JA and expression of JA-responsive genes have been associated with an increase in response to pest feeding [25], pathogen infection, or tissue damage [26] while exogenous application of JA has been associated with expression of defence-related genes [26]. These host defence-related genes may confer resistance, tolerance or susceptibility to pathogens and herbivores. The exact mechanisms, which herbivores use to leverage SA-JA antagonism, are still poorly understood. This is especially the case in cassava where detailed transcriptome studies are lacking. There is a wide knowledge gap in our understanding of processes and pathways involved during mealybug-cassava interaction. Transcriptome analysis promises to enhance our understanding of this interaction and could lead to the identification of important candidate genes.

There are, however, a few transcriptome studies that have been reported in cassava including the analysis of the transcriptome of landraces infected with *South African cassava mosaic virus* [27] using the ABI SOLiD NGS platform. A transcriptome profile of low temperature-treated cassava apical shoots demonstrated dynamic response to abiotic cold stress treatment [28] resulting in the expression of MYB and WRKY transcription factors and CYP450 and MAP kinases, which are known to play a role in response to abiotic stress. The expression of the aforementioned genes has also been reported during pathogen and insect attack [8, 29–31] suggesting their involvement in plant defence. In the current study, transcriptomes were compared between a susceptible (P40/1) and a resistant (AR23.1) cassava genotype pre- (0 hours) and post- (24 and 72 hours) mealybug (*P*. *manihoti*) feeding. Differentially expressed genes were mapped to the castor bean (*Ricinus communis*) reference genome in KEGG to predict gene pathways that were involved in *P*. *manihoti* feeding. Genes that were involved in plant-pathogen interactions and plant hormone signal transduction in early response to cassava mealybug feeding were profiled, and compared between AR23.1 and P40/1. We identified specific candidate defence-related genes induced only in the insect resistant cassava genotype AR23.1 and not in the susceptible genotype P40/1. These findings could contribute to future development of new resistant cassava cultivars using gene editing methods such as CRISPR [32].

## Materials and methods

### Mealybug collection and maintenance

Mealybugs were scouted randomly from cassava growing farms at Makhathini, KwaZulu-Natal province, Malelane and Bushbuckridge, Mpumalanga province. The collected mealybugs were transported to the Agricultural Research Council (ARC)–Vegetable and Ornamental Plants Institute (VOPI) and maintained as independent colonies on cassava plants under controlled conditions in the glasshouse. A sample of each mealybug collection was preserved in 100% ethanol and stored at room temperature for morphological and molecular identification. Mealybug specimens were prepared for morphological and taxonomic identification as previously described [33]. The South African Authority does not require any permission for scouting and/or collection of pests of any kind either from open fields or from farmers’ fields. The current study did not involve endangered or protected species.

### Confirmation of mealybug identity

Total DNA was extracted from individual intact specimens using the blood and body fluid protocol of the DNeasy DNA extraction kit (Qiagen, Valencia, CA), with modifications according to Sethusa et al. [16]. DNA concentrations were quantified using Qubit (Invitrogen/Life Technologies Inc., Carlsbad, CA, US) and verified on a 1% agarose gel. Barcoding primers of the 28S ribosomal RNA (rRNA) region, s3660 (F- 5’GAGAGTTMAASAGTACGTGAAAC3’) [34] and 28S (R- 5’TCGGAAGGAACCAGCTACTA3’) [35]; and the 18S rRNA region, 2660 (F- 5’CTGGTTGATCCTGCCAGTAG3’) [36] and 18S (R- 5’CCGCGGCTGCTGGCACCAGA3’) [37] were used to amplify genomic regions of mealybug samples. Gene fragments were amplified using a touchdown protocol that included initial denaturation at 94°C for 5 min followed by 14 cycles of 94°C for 30 sec, 56°C for 30 sec and 72°C for 2 min, with 1.0°C decreasing temperature from 56 to 42°C for 30 sec every cycle. The remaining 18 cycles were performed at 94°C for 30 sec, 42°C for 30 sec and 72°C for 2 min, with the final elongation at 72°C for 7 min. PCR products (700 bp) were purified using the QIAquick PCR purification kit (Qiagen, Valencia, CA). After PCR amplification, the products were verified on 1% agarose gel electrophoresed in 1xTAE buffer.

Sequencing of PCR products was outsourced to Inqaba Biotec (Gauteng, South Africa). Resultant sequences were assembled using Pregap4 and Gap4 programs within the molecular biology software STADEN package [38]. Consensus sequences were subjected to BLASTn on NCBI (www.ncbi.nlm.nih.gov/BLAST) to retrieve known homologous sequences as species identification references. The molecular evolutionary genetics analysis package (MEGA v.6.0) was used for multiple sequence alignment and phylogenetic analysis. Non-parametric maximum likelihood (ML) bootstrapping with heuristic searches of 10,000 replications was performed to assess branch support in phylogenetic trees generated. Values of 95% or larger were regarded as evidence that the groupings were of the same species, while values of 70% - 94% were regarded as groupings of a related species.

### Cassava collection, multiplication and hardening

Cassava genotypes AR23.1 and P40/1 were sourced from the Agricultural Research Council-Vegetable and Ornamental Plant Institute (ARC-VOPI, South Africa) and multiplied through tissue culture (Table 1) in Murashige and Skoog (MS) medium [39] in a growth room at 21°C (16 h light and 8 h darkness) until the plants developed roots and leaves (7 weeks). *In vitro* plantlets were later transferred to 20 cm diameter pots under glasshouse conditions at 27 ± 2°C day and night temperature, grown in a medium mix of 60% topsoil, 20% sand and 20% vermiculite. Plants were watered once daily, and supplemented with nutrients (Multifeed, Makro, SA) for a period of seven weeks.

**Table 1:** Cassava genotypes selected for mealybug infestation and transcriptome experiments.

Genotype	Origin	Source	Other information
AR23.1	CIAT, Colombia	ARC-VOPI	Stay green, grow fast in vitro and in glasshouse, multiple disease and green mite resistance
P40/1	ARC, South Africa	ARC-VOPI	No known resistance

### Mealybug infestation experiments and scoring

Plant leaves that were seven weeks old were infested with the third stage instar mealybugs. Nine plants were divided into groups of 3, each representing a biological replicate. Plants were prepared separately for both phenotypic evaluation and RNA-sequencing under the same conditions. Two fully expanded leaves (second leaves from the bottom) were infested with 15 mealybugs by gently brushing the insects on the leaves using a fine paintbrush. Non-infested controls were plants from which leaf tissues were harvested at zero-time point prior to infestation. Mealybugs were removed from the infested leaves using a paintbrush before harvesting plant tissues for RNA-sequencing. Leaves infested with mealybugs or mock-infested (leaves were wiped with sterile distilled water using a sterile paintbrush) were harvested at 24 and 72 hours (Table 2).

**Table 2:** Description of experimental samples.

Tissue	Description	Genotype	RNA-seq dataset
Non-Infested tissue harvested at 24 h	Non-infested mock	AR23.1	AR23-24-mock
Infested tissue harvested at 24 hpi	Experimental tissue	AR23.1	AR23-24-inf
Non-Infested tissue harvested at 72 h	Non-infested mock	AR23.1	AR23-72-mock
Infested tissue harvested at 72 hpi	Experimental tissue	AR23.1	AR23-72-inf
Non-Infested tissue harvested at 24 h	Non-infested mock	P40/1	P40-24-mock
Infested tissue harvested at 24 hpi	Experimental tissue	P40/1	P40-24-inf
Non-Infested tissue harvested at 72 h	Non-infested mock	P40/1	P40-72-mock
Infested tissue harvested at 72 hpi	Experimental tissue	P40/1	P40-27-inf

Leaf tissues were pooled from each plant per replicate (6 leaves per biological replicate), snap frozen in liquid nitrogen and stored at -80 ^o^C. The plants were further monitored weekly post infestation (7, 14, 21, 28 and 35 dpi) to assess and record the extent of damage and the number of mealybugs per surviving plant. Phenotypic data variations between the replicates, different time points and genotypes were detected using ANOVA [40].

Screening and scoring were done according to the method of the Collaborative Study of Cassava in Africa [41] on the 7^th^, 14^th^, 21^st^ 28^th^, and 35^th^ days post-infestation by comparing mealybug-infested with mock-infested leaves. Symptom severity was evaluated per plant on a scale of 0 (resistant, no observable symptoms) to 4 (a candlestick-like appearance, internode length reduced, curved/complete defoliation of young portion of shoots, ≥200 mealybugs and severe symptoms). Plants that had 200≤mealybugs≥100 with moderate symptoms were scored as moderately susceptible (score 3) while those with 100≤mealybugs≥50 were scored as moderately resistant/tolerant (score 2). Plants with less than 50 mealybugs, and slight symptoms were scored as partially resistant (score 1).

### RNA isolation and quantification

Leaf tissues (100 mg) from infested and non-infested (mock) plants from each biological replicate (6 leaves from 3 plants were pooled) and time point were ground in liquid nitrogen. RNA was isolated using RNeasy Plant Mini kit (Qiagen, Valencia, CA) according to the manufacturer’s instructions. RNA was digested with DNase using the RNase-Free DNase kit (Qiagen, Valencia, CA), subsequently eluted in 50 μl RNase-free water, and stored in -80 **°**C until ready for use. RNA concentration was quantified using a Qubit® (Invitrogen/Life Technologies Inc., Carlsbad, CA, US), and agarose gel electrophoresis to view RNA quality. After quantifying concentrations using Qubit, all RNA samples were adjusted to the same concentration for reverse-transcription reactions and sequencing.

### Library preparation and RNA sequencing

Total RNA was purified by the removal of rRNA using magnetic kit (www.epicentre.com) to produce enriched mRNA that was prepared for sequencing using a TruSeq Stranded mRNA Sample Preparation Kit (Illumina, San Diego, CA, USA). First strand cDNA was synthesized using reverse transcriptase and random hexamers followed by the second strand synthesis. The cDNA fragments were 3′ adenylated followed by adapter ligation with RNA adapter indexes. PCR amplification was performed to enrich purified cDNA template. The cDNA concentration of the library was determined using a Qubit® (Invitrogen) according to the manufacturer’s instructions, and the library was further validated on a 1% 1xTAE agarose gel. Library dilutions were prepared and loaded on a cBOT system (Illumina, San Diego, CA) for cluster generation and further sequenced using Illumina HiSeq 2500. Each biological replicate (3 per time point) was sequenced separately for each genotype AR23.1 and P40/1 at 24 and 72 hpi as well as for mock. An RNA-seq workflow for the methods is shown in S1 Fig.

## Bioinformatics analyses

### Read mapping and gene expression quantification

The quality of sequencing data was assessed using the FastQC v0.10.1 tools [42]. Data of the biological replicates of each genotype was calculated to determine the positive correlation within the same time-point using MS Excel. Since the three biological replicates had no significant variation between them, data from three biological replicates were pooled together per time-point for each genotype before analysis of differentially expressed genes. Sequences were trimmed for quality and adaptors removed using Trimmomatic v0.30 software [43]. The sequences used for further analysis had a Phred score with a confidence level above 20. Trimmed transcript sequences were mapped to the annotated cassava reference genome (Mesculenta_305_v6.1) and quantified using Tophat/Cufflinks v2.0.11 pipeline [44, 45]. Differential gene expression in response to mealybug infestation was analysed using the Cuffdiff tool [45]. Gene expression counts were normalized using fragments per kilobase of exon per million mapped reads (FPKM). Differentially expressed genes (DEGs) were determined with a log2 fold change > 2.0 cut-off and an absolute *P*-value of ≤ 0.05. Differentially expressed genes were assigned functions manually using the Mesculenta_305_v6.1 annotation file, according to their gene ID, and compared to public databases from RNA-seq analysis post infestation with herbivores [23, 29, 30, 46, 47].

Gene ontology (GO) analysis was conducted on the differentially expressed genes using InterProScan (http://www.ebi.ac.uk/Tools/pfa/iprscan/) with default parameters. The GO terms associated with each DEG were then obtained for describing biological processes, molecular functions and cellular components. InterProScan output file was input into BGI WEGO program and GO annotations were plotted (http://wego.genomics.org.cn) [48]. The raw data was submitted to the NCBI Small Read Archive (accession number SRP058748).

### Pathway analysis

Pathway analysis was performed using the Kyoto Encyclopedia of Genes and Genomes (KEGG) annotation service [49, 50]. KEGG Ontology (KO) terms were assigned to DEGs using the *M*. *esculenta* annotation file (www.phytozome.net). Genes that were assigned KO terms were then mapped to KEGG pathway and significantly enriched terms further identified in comparison with the castor bean (*Ricinus communis*) genome background.

### RT-qPCR validation of DEGs

Nine (9) candidate defence-related genes (Table 3) were selected from the DEGs for validation using quantitative PCR. Primer pairs were designed using CLC Bio Genomics Workbench v6.5, with the amplicon length less than 200 bp and melting temperature between 50–60 **°**C. GTP binding (GTPb) [51] was used as a reference gene for the normalization of the RT-qPCR data.

**Table 3:** Primer sequences of selected DEGs used for real-time quantitative reverse transcription-polymerase chain reaction (RT-qPCR).

Genes	Primer names	Forward (5’-3’)	Reverse (5’-3’)	Product size
1. *Ubiquitin 10*	UBQ10	tgcatctcgttctccgattg	gcgaagatcagtcgttgttgc	166 bp
2. *GTP binding*	GTPb	gttgccttcttttgcgtttct	gcaatttgatccgttttccat	114 bp
3. *2-Oxyglutarate (2OG) and FE(II)-dependent oxygenase superfamily protein*	2OG-FE(II)	ggagttgaggttgctgaaga	ggaaggaggagaagaagaaga	146 bp
4. *HSP20-like chaperones superfamily protein*	HSP20	aaaaacccacaagcccaa	ctctcactttctcttcatcttc	190 bp
5. *Myb domain protein 4*	MYB	ggtgagggtggtttaagt	ctctagatttgcgtttgatg	161 bp
6. *Glycosyl hydrolases family 32*	GH32	gattaggcgttgaagaag	gaacaggagctgaagcta	145 bp
7. *Cytochrome P450*, *family 94*, *subfamily B*, *polypeptide 3*	CYP450	actcccacttatcctacac	caacgaaaccacaaccaa	120 bp
8. *Alpha/beta-hydrolases protein family*	Alpha/B	cactctctcttctacttcttct	ttttcccttgtggccctt	179 bp
9. *GDSL-like Lipase/Acylhydrolase family protein*	GDSL	ggttttgtaatggagtgg	tattttgggtgtgagagg	176 bp
10. *Terpene synthase 21*	Terp	tcttcttcttctcctttcctc	tccccacttacaatcccc	141 bp
11. *Gibberrelin 2-oxidase 6*	Gibb	tgcatgattcctgactagtc	catggatcagtctagaatg	150bp

Reverse transcription (RT) of RNA was achieved using iScript Advanced first strand cDNA synthesis kit (Bio-Rad Laboratories Inc., Hercules, CA, USA) in a total reaction volume of 20 μl containing 1 μg of total RNA according to the manufacturer’s instructions. Complementary DNA (cDNA) concentrations were quantified using Qubit® fluorometer, and equal concentrations (40ng) were prepared for RT-qPCR reactions. Quantitative RT-qPCRs were performed in a Roche LightCycler® 96 System (Roche Applied Science, US) using the KAPA SYBR FAST Universal qPCR kit (KAPA Biosystems, SA) according to the manufacturer’s instructions. Each qPCR reaction was accomplished in triplicate with negative controls. Fluorescence values were acquired at the end of the elongation phase. A melting curve analysis was performed after the final PCR cycle to verify primer specificity. The efficiency of each primer pair was checked for each reference gene using the linear regression method. Transcript levels were estimated using the relative quantification method, as described by Livak and Schmittgen [52] and Pfaffl [53] by calculating the ratio of relative expression from the efficiency (E) and threshold cycle (C_t_) values of an unknown sample versus a control sample. The stability of the reference genes across all samples was determined using BestKeeper statistical algorithm [54].

## Results

### Identification of mealybugs

Morphological examination and comparison using the species taxonomic key pointed to the presence of one species of the *Phenacoccus* genus, *Phenacoccus manihoti* (Matile-Ferrero). Important diagnostic features, such as the position of the pores, ducts and setae on the dorsum, matched the distribution patterns as depicted in the Williams & de Willink [33] illustration, confirming the morphological identity of mealybugs in South Africa.

**Fig 1:**
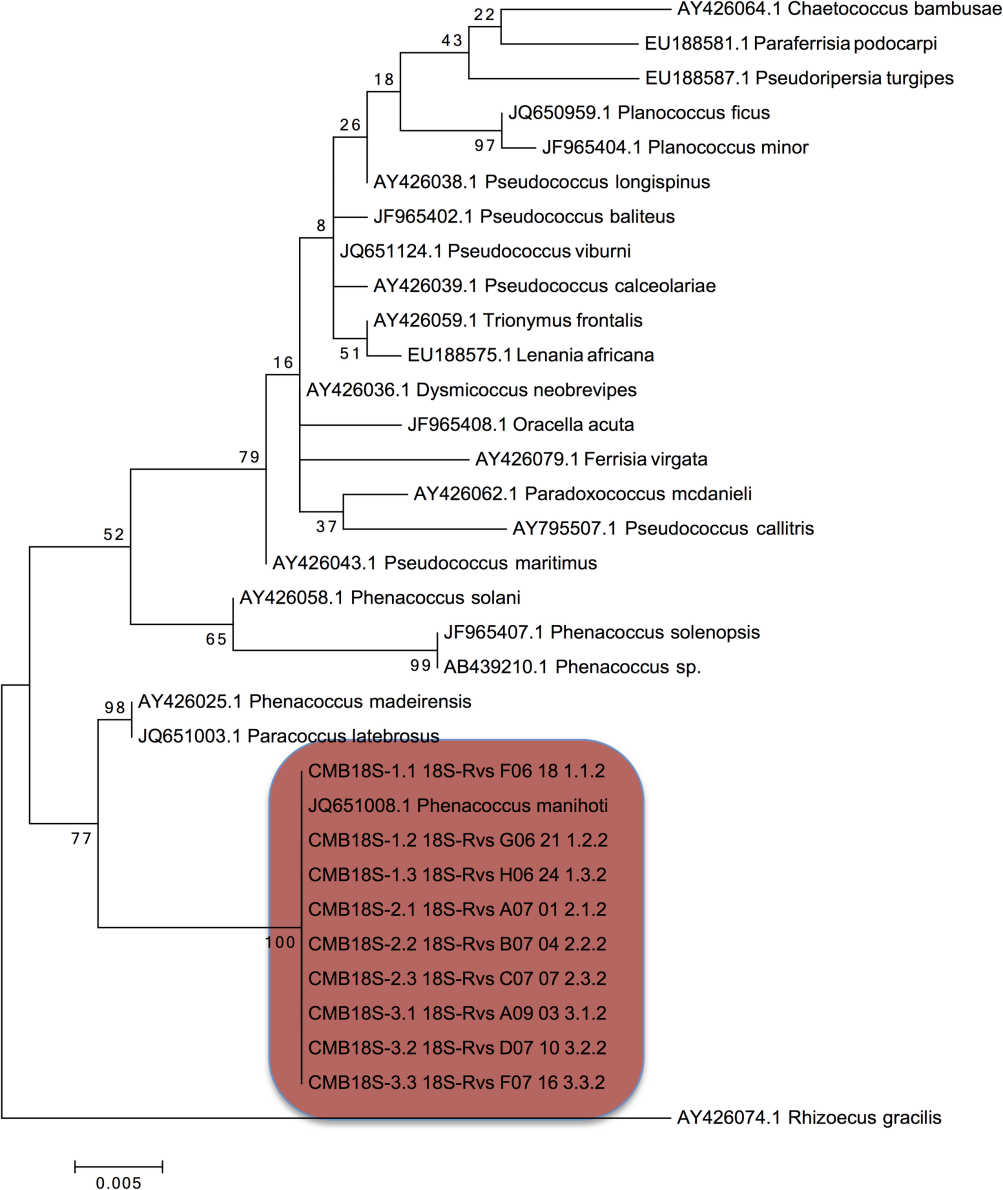
Classification of the South African mealybug species collected from cassava using 18S rRNA. Phylogenetic tree of *Phenacoccus* species derived from the maximum likelihood analysis of 18S rRNA sequences. Mealybug samples collected in the current study (colour-shaded) clustered with *Phenacoccus manihoti* 18S RNA sequence (Genbank accession number JQ651008.1) confirming their identity. Bootstrap values were calculated from 10 000 replicates.

**Fig 2:**
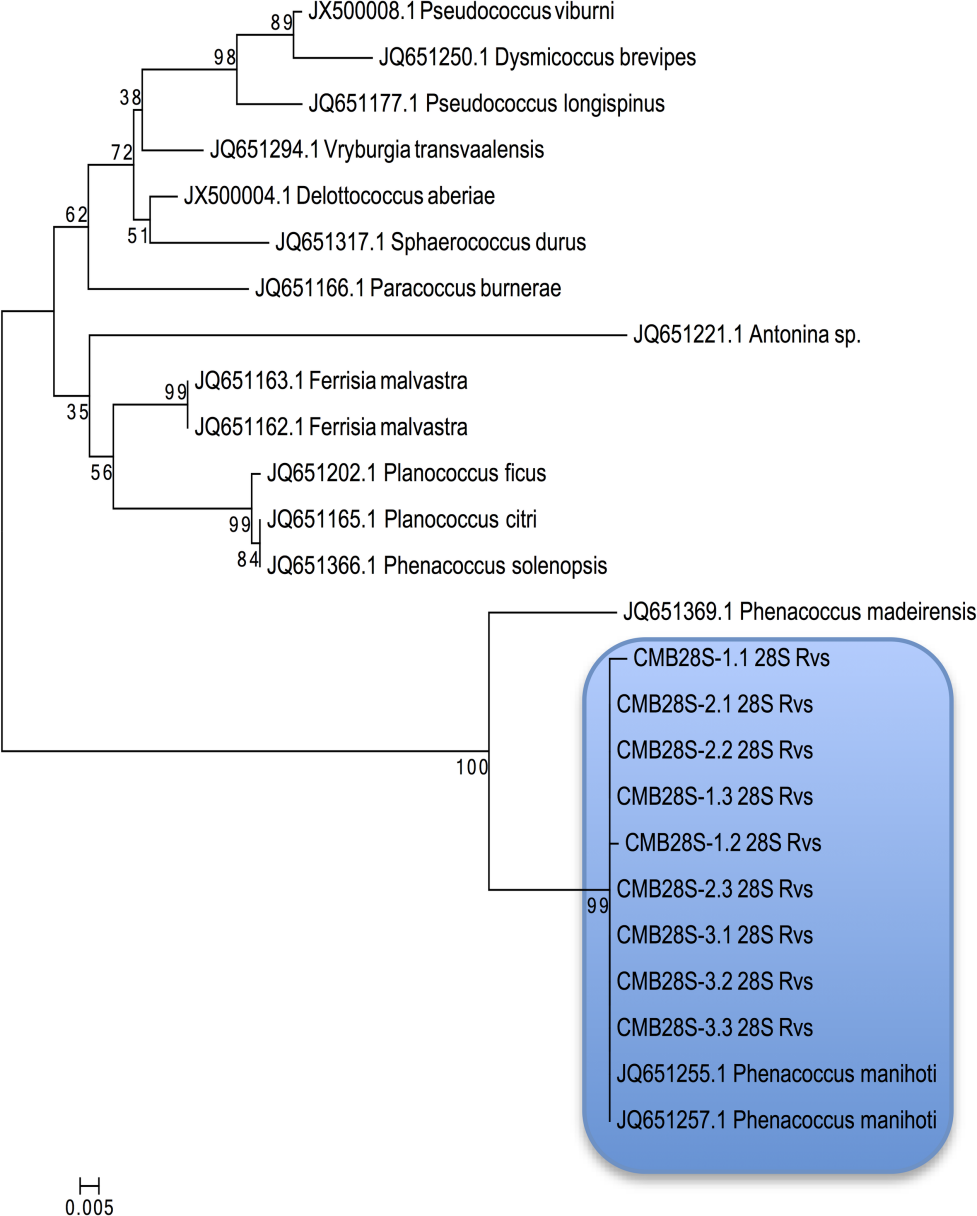
Classification of the South African mealybug species collected from cassava using 28S rRNA. Phylogenetic tree of *Phenacoccus* species derived from the maximum likelihood analysis of 28S rRNA sequences. Mealybug species highlighted here also clustered with *Phenacoccus manihoti* when 28S RNA sequences (Genbank accession number JQ651255.1 and JQ651257.1) were used to draw the phylogenetic tree. Bootstrap values were calculated from 10 000 replicates.

Molecular analysis further confirmed the morphological identity of the sampled mealybug species as *P*. *manihoti*. The amplification of 18S and 28S rRNA regions of the sampled mealybug species resulted in the expected 700 bp products, which were subsequently confirmed through Sanger sequencing. Both the 18S and 28S rRNA generated sequences of the nine individual mealybug samples clustered with known *Phenacoccus manihoti* sequences (JQ651008.1, JQ651255.1 and JQ651257.1) that had been retrieved from the Genbank with 100% (Fig 1) and 99% (Fig 2) similarity respectively. Madeira mealybug (*Phenacoccus madeirensis*) was confirmed to be the closest relative to *P*. *manihoti* in both 18S and 28S rRNA analysis (Figs 1 and 2). Consensus sequences were submitted to the NCBI GenBank accession numbers KY271370-KY271372 and MG906880- MG906885 (18S sequences); and KY271373-KY271375 and MG910449- MG910454 (28S sequences).

### Cassava phenotypic responses to mealybug infestation

**Fig 3:**
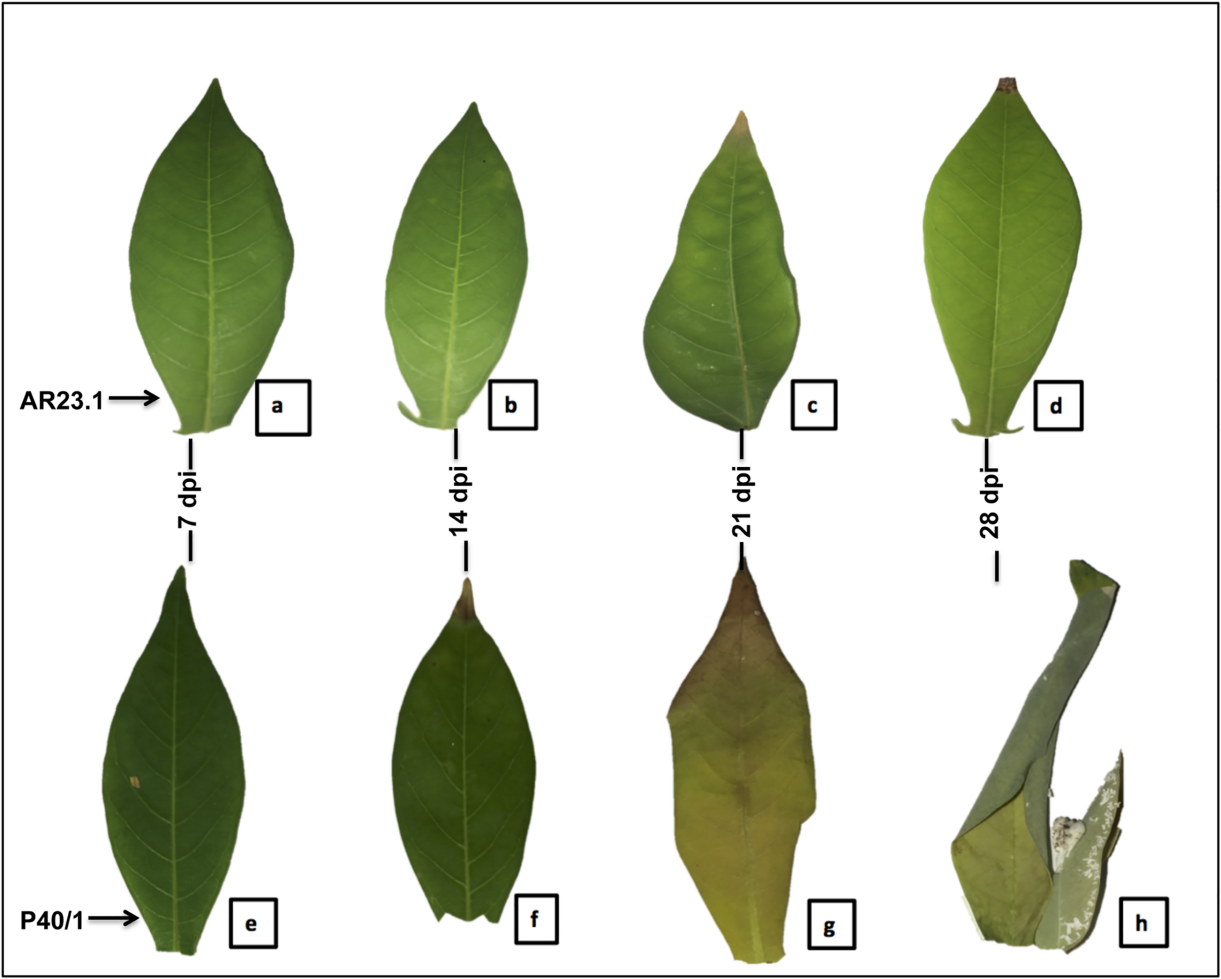
Mealybug-induced symptoms on the plants. Cassava genotypes AR23.1 (a-d) and P40/1 (e-h) plants infested with mealybugs (*P*. *manihoti*) in a glasshouse at ARC-VOPI, South Africa (R753 25° 59″ S 28° 35″ E). A clear difference on the effect of mealybug across the two genotypes is observed from 21 days post infestation (dpi). The only observable symptom on AR23.1 leaves at 28 dpi is yellowing on the tips (Fig 3D) while P40/1 leaves soften and show signs of yellowing and curling at the edges at the same time point (Fig 3H).

From the phenotypic evaluation, AR23.1 was regarded as partially resistant and P40/1 as susceptible (Table 4). Leaves of infested AR23.1 genotype were asymptomatic up to 14 days post infestation (dpi) (Fig 3A and 3B) but subsequently showed mild morphological changes around 21 (Fig 3C) and 28 dpi (Fig 3D). In contrast, P40/1 showed distinct symptoms with extensive wilting and chlorosis by 21 dpi (Fig 3G). While the AR23.1 plants continued to regenerate new leaves despite active colonization by the insects, P40/1 plants did not regenerate any new leaves and subsequently wilted and died (Fig 3H).

**Table 4:** Scoring of AR23.1 and P40/1 cassava genotypes for phenotypic responses to cassava mealybug (CMB) infestation at 7, 14, 21, 28 and 35 dpi.

	AR23.1		P40/1	
Days post infestation (dpi)	CMB counts	Symptoms	CMB counts	Symptoms
7 dpi	< 50 CMB	No visible symptoms	> 50 CMB	Slight insect nodules on leaves
14 dpi	< 50 CMB	Plant green with slight insect nodules	± 100 CMB	Moderate insect nodules
21 dpi	± 50 CMB	Moderate insect nodules and slight change of leaf colour	> 150 CMB	Change in leaf colour
28 dpi	≥ 50 CMB	Dying of old damaged leaves	> 200 CMB	Curled leaves, severe reduction of leaf tissue, Plant dying
35 dpi	< 50 CMB	Slight loss of leaf tissue	> 200 CMB	Death of a plant
**Score**	**1 –Partially resistant**		**4—Susceptible**	

### Gene expression of cassava genotypes in response to mealybug infestation

A detailed account of all sequencing reads generated per biological replicate is provided in S1 Table. High quality reads were generated, resulting in the retention of at least 85% of the sequences after trimming (S1 Table). There was also good correlation between reads from the different biological replicates of each genotype, with correlation values ranging from R^2^ = 0.991–0.999 (S2 Fig) for AR23.1 to R^2^ = 0.998–0.999 (S3 Fig) for P40/1. Given the significant correlation across replicates, the reads were pooled from the three biological replicates at each time point for each sample before mapping reads to the reference genome. Table 5 shows a summary of pooled reads per time point for each genotype before and after trimming. On average, 84% of the trimmed reads could be mapped to the cassava reference genome (Mesculenta_305_v6.1) (Table 5) using Tophat/Cufflinks [44, 45].

**Table 5:** Summary of raw and trimmed reads generated by Illumina HiSeq 2500. Reads are presented per time point after pooling across the three biological replicates of AR23.1 (resistant) and P40/1 (susceptible); and subsequently mapping to the cassava reference genome (Mesculenta_305_v6.1).

RNA-seq dataset	No. of raw reads	Trimmed reads	% Trimmed reads	Mapped reads	Mapped reads/million sequence reads
AR23-24-mock	81, 490,264	78,829,123	96.7	70,228,998	890,901
AR23-24-inf	85,904,324	82,499,207	96.0	68,587,064	831,366
AR23-72-mock	120,666,228	112,979,340	93.6	85,831,373	759,708
AR23-72-inf	116,142,504	104,578,982	90.0	90,357,903	864,015
P40-24-mock	88,204,836	85,273,331	96.6	76,921,462	902,057
P40-24-inf	84,905,130	79,802,477	94.0	66,220,010	829,798
P40-72-mock	98,680,132	91,365,796	93,3	74,305,354	813,273
P40-27-inf	95,190,544	90,807,902	95,4	77,618,821	854,758

**Fig 4:**
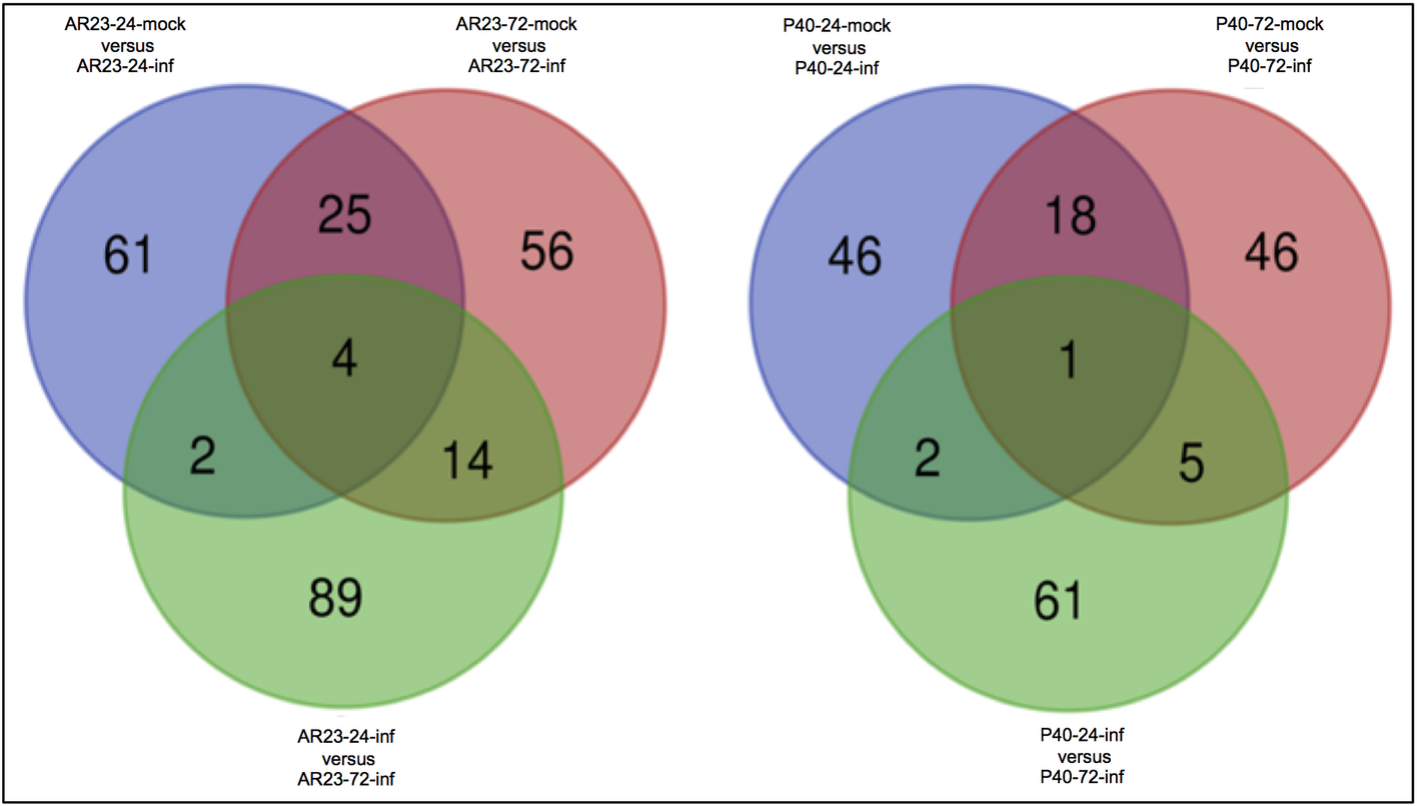
Venn diagrams of differentially expressed genes (DEGs). A comparison of DEGs that were common across different time points (24 and 72 hours compared to non-infested mock) in AR23.1 and P40/1 post-infestation with *P*. *manihoti*. The Venn diagrams represent an average across all three biological replicates at respective time points. Only transcripts with a minimum of log2 fold change and P ≤ 0.05 were included in the data.

**Fig 5:**
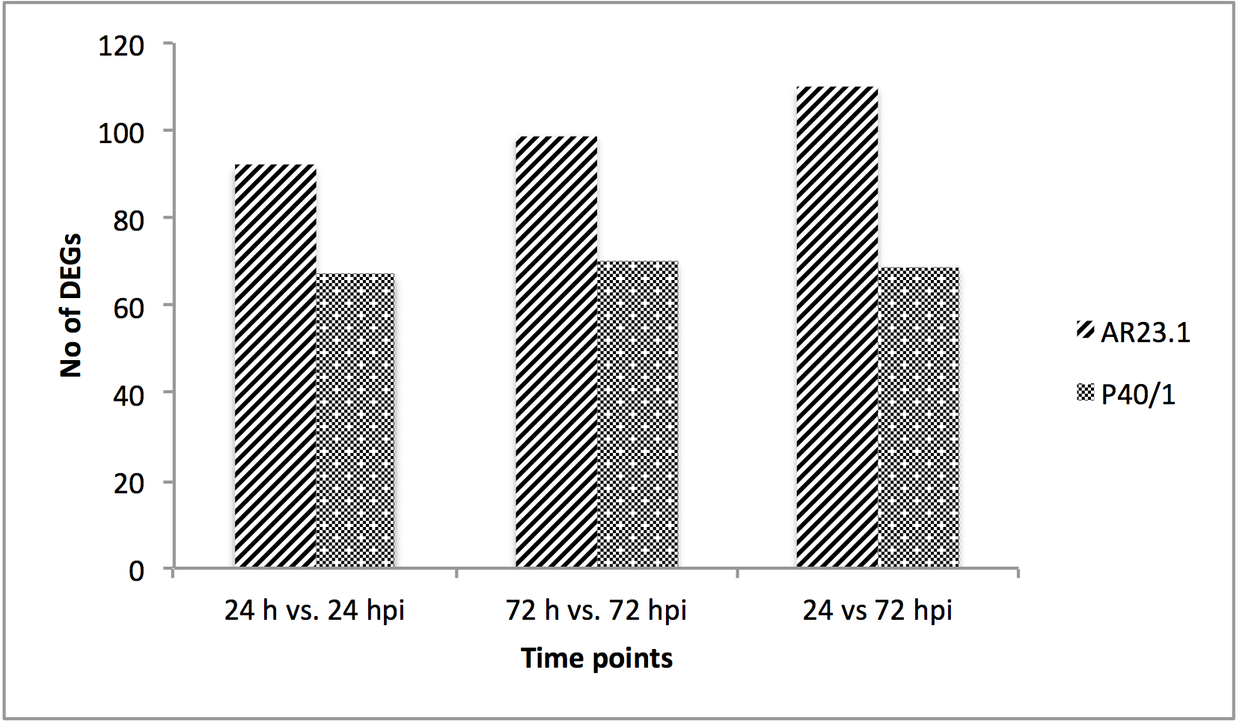
Number of DEGs post-infestation with mealybug. Compared to non-infested mock plants, the number of DEGs increased after mealybug infestation from 24 to 72 hours for AR23.1 and P40/1.

A total of 301 and 206 transcripts from AR23.1 and P40/1 respectively were differentially expressed (log2 fold change and *P ≤ 0*.*05*) at various time points following mealybug infestation (Table 6). In both genotypes, mealybug infestation resulted in a higher number of down-regulated than up-regulated genes between mealybug-infested and mock tissues (Table 6), except in P40/1 between infested leaves at 24 and 72 hours of mealybug infestation, where up-regulated genes were more than the down-regulated genes (Table 6). Venn diagrams revealed that most of the DEGs observed were uniquely expressed at specific time points and that there were less common DEGs between different time points in both genotypes (Fig 4). Of all the DEGs identified, only 4 and 1 were common at 24 and 72 hours for genotype AR23.1 and P40/1, respectively (Fig 4). There was a steady increase in the number of DEGs with increase in time post infestation (Fig 5) suggesting an increase in mealybug defence response with progress in infestation.

**Table 6:** An overview of the number of up-regulated and down-regulated differentially expressed genes identified between non-infested mock and mealybug infested leaves at 24 and 72 hours. In P40/1 the number of down-regulated DEGs between the infested tissues at 24 hpi versus 72 hpi was lower compared to the number of up-regulated DEGs.

Dataset 1	Dataset 2	Time-points	Up-regulated	Down-regulated	Total
AR23-24-mock	AR23-24-inf	24 h versus 24 hpi	38 (41%)	54 (59%)	92
AR23-72-mock	AR23-72-inf	72 h versus 72 hpi	42 (42%)	57 (58%)	99
AR23-24-inf	AR23-72-inf	24 hpi versus 72 hpi	33 (30%)	77 (70%)	110
		**Total**	**113 (38%)**	**188 (62%)**	**301**
P40-24-mock	P40-24-inf	24 h versus 24 hpi	31 (46%)	36 (54%)	67
P40-72-mock	P40-72-inf	72 h versus 72 hpi	27 (39%)	43 (61%)	70
P40-24-inf	P40-72-inf	24 hpi versus 72 hpi	38 (55%)	31 (45%)	69
		**Total**	**96 (47%)**	**110 (53%)**	**206**

### Transcriptional changes in AR23.1 in response to mealybug infestation

**Fig 6:**
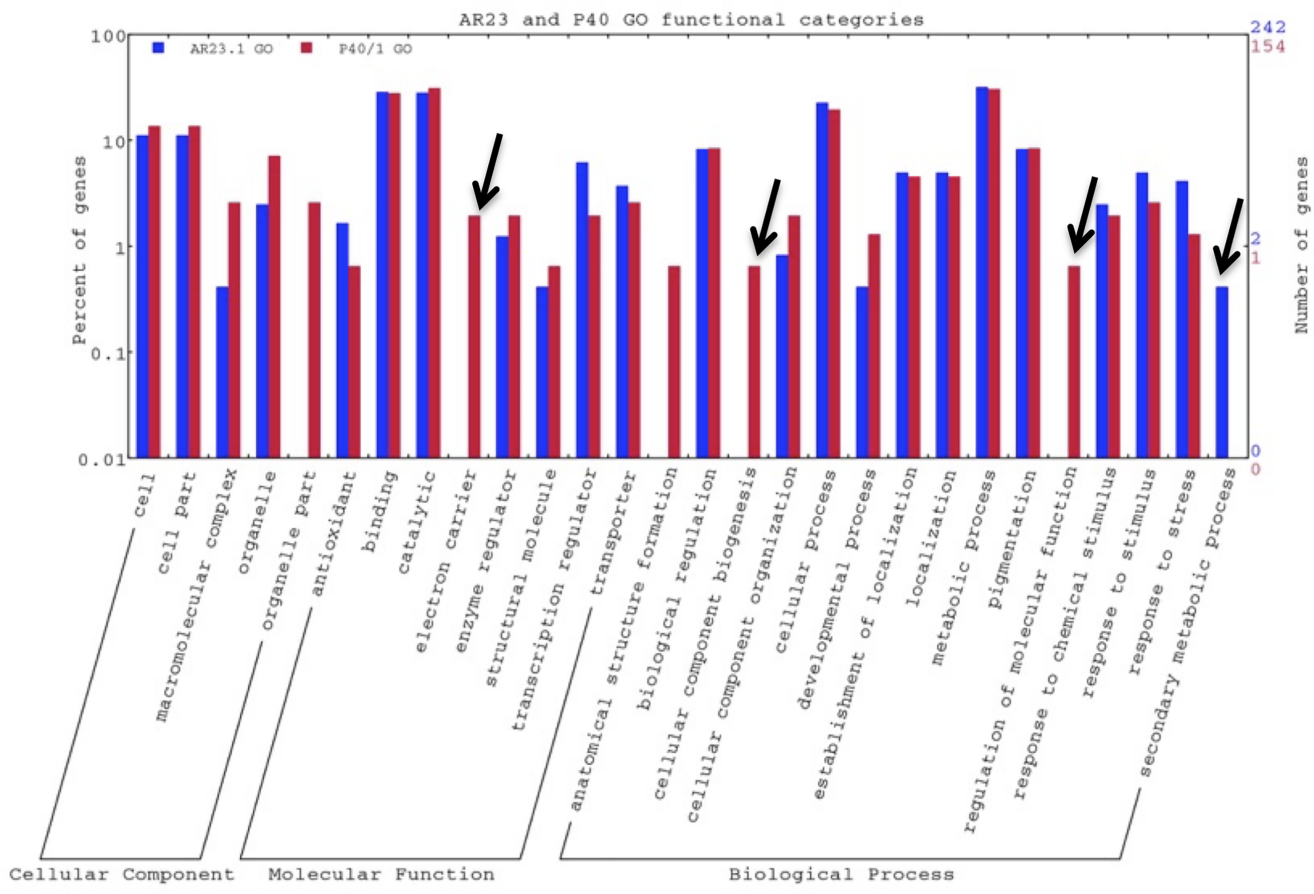
Analysis of enriched GO terms. Functional categorization of all unique differentially expressed genes based on biological processes, cellular components and molecular functions for AR23.1 and P40/1 genotypes. Significant differences observed between the DEGs of the two genotypes are highlighted using the pointing arrows.

Out of the 301 differentially expressed genes (DEGs) identified (Table 6) in AR23.1, 242 were unique (S2 Table). We assigned GO terms for 132 out of the 242 unique DEGs (55%) for describing biological processes, cellular components and molecular functions (Fig 6). Under the GO functional categorization, DE genes were highly represented in the cellular process, metabolic process, response to stimulus and response to stress in the biological process category. Binding and catalytic functions were over-represented within the molecular function category while the most abundant DE genes under cellular component category were the cell and cell part (Fig 6).

We observed abundance of pathogenesis-related (PR) proteins among the differentially expressed proteins in AR23.1, most of which were classified as resistance (R) receptor like detection and signalling proteins (S2 Table). We also detected two Leucine-rich repeat receptor-like protein kinase (LRR-PK) family proteins (Manes.03G048300, Manes.01G142200) that were up-regulated within 24 hours of mealybug infestation, and a third one (Manes.01G129500) that was up-regulated at 72 hours of mealybug infestation (S2 Table). Most of the PR proteins appeared to be induced within 24 hours of mealybug infestation including an HSP20-like chaperone superfamily protein (Manes.02G124600), and MYB domain proteins 4 (Manes.01G147500) and 55 (Manes.01G235800). There was evidence of reduced expression by 72 hours of mealybug infestation demonstrating a decreased expression of these proteins after the first 24 hours of infestation with mealybugs.

However, we also observed both up- and down-regulation of some of the pathogenesis-related proteins at 24 hours of mealybug infestation including WRKY DNA-binding protein 56 (Manes.01G252200) and WRKY family transcription factor (Manes.01G273700). Other known insect-defence related proteins, such as Cytochrome P450 family 339 genes (Manes.01G200000, Manes.15G189500, Manes.15G154400, Manes.01G178000, Manes.01G264600, Manes.10G146700, Manes.03G138800, Manes.08G047000, Manes.02G071200) as well as kunitz trypsin inhibitors (Manes.01G126300), were either up- or down-regulated at both 24 and 72 hours of mealybug infestation (S1 and S2 Tables).

**Fig 7:**
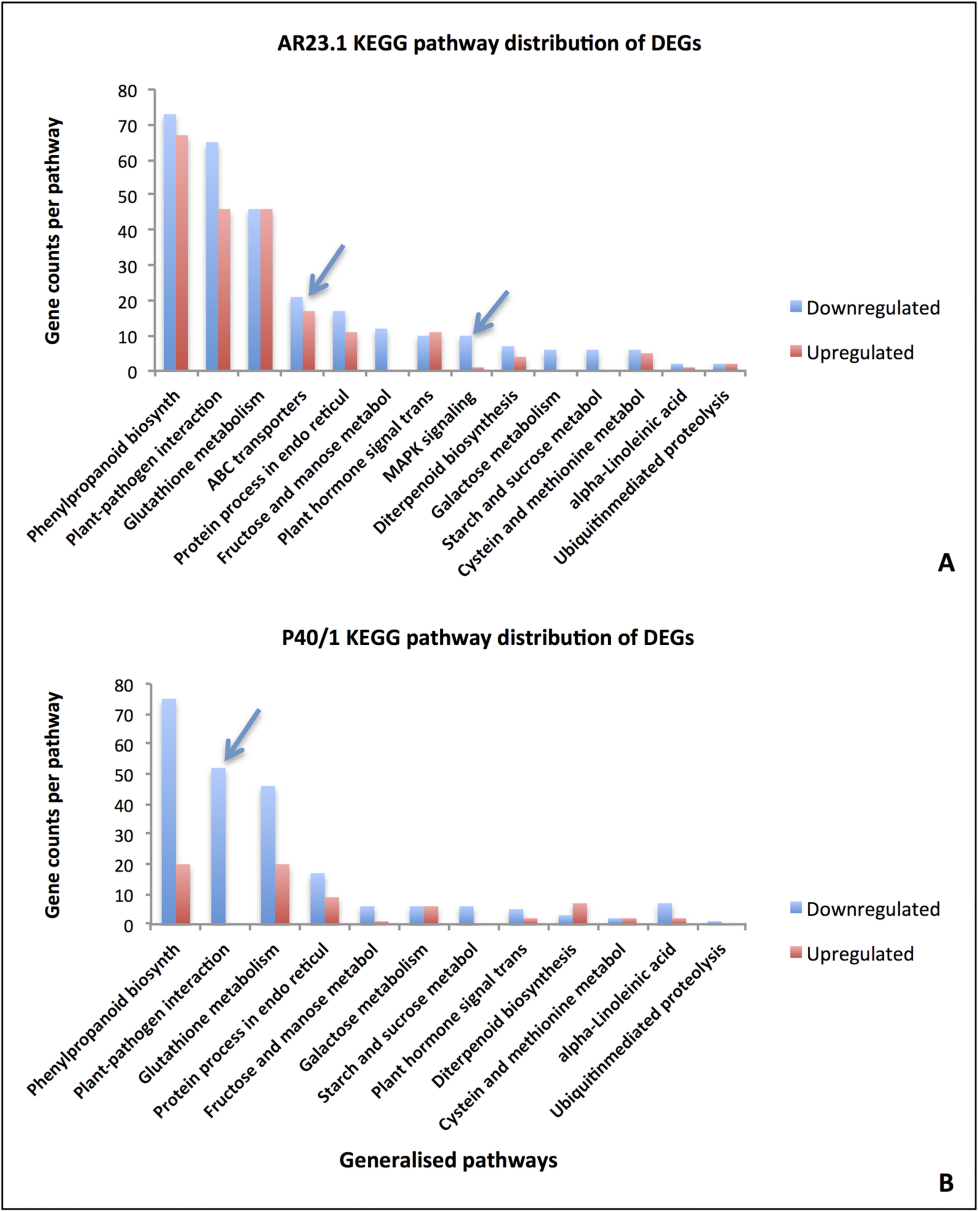
KEGG classification. The KEGG pathway distribution of DEGs in AR23.1 (A) and P40/1 (B) in response to cassava mealybug infection using castor bean (*Ricinus communis*) as the reference. The graph represents the 14 most enriched pathways, and the number of genes per pathway. Significant differences observed between the two genotypes are pointed with arrows.

Metabolic pathway analysis, using the KEGG database, revealed that 73 out of 244 unique DEGs (30%) identified from AR23.1 mapped to 14 KEGG pathways (Fig 7A). There were more down-regulated than up-regulated genes in almost all of the KEGG pathways mapped (Fig 7A), although a high percentage of genes could not be mapped due to a lack of KO terms.

### Transcriptional changes in P40/1 in response to mealybug infestation

Out of the 206 DEGs identified (Table 6) in P40/1, 154 were unique (S3 Table). We assigned GO terms for 86 out of the 154 unique DEGs (56%) for describing biological processes, cellular components and molecular functions (Fig 6). GO functional categorization was over-represented in the cellular and metabolic process, and response to stress in the biological process category, binding and catalytic in the molecular function category, and cell and cell part in the cellular component category (Fig 6). However, genes under regulation of molecular function, electron carrier and organelle part categories were specifically enriched in the P40/1 genotype in comparison with AR23.1.

Similar to the genotype AR23.1, we observed up-regulation of the Leucine-rich repeat receptor-like protein kinase family protein (Manes.02G150200) and leucine-rich repeat transmembrane protein kinase family protein (Manes.03G048300) within 24 hours of mealybug infestation. Other pathogenesis related (PR) proteins such as IAA-leucine resistant (ILR)-like 2 (Manes.01G196200) genes were only up-regulated at 72 hours of mealybug infestation. The same disease resistance gene (Manes.02G205700) belonging to the TIR-NBS-LRR class of R genes, that was found to be down-regulated at 72 hours of mealybug infestation in a resistant genotype AR23.1 (S2 Table), was also down-regulated in susceptible genotype P40/1 (S3 Table) at the same time point. Moreover, two other disease resistance family proteins (TIR NBS LRR; Manes.11G156500, Manes.14G165100) were also down-regulated in mealybug-infested tissues harvested at 24 hours compared with mealybug infested tissues harvested at 72 hours. Since the TIR NBS LRR R-genes are involved in the division of signalling pathways leading to disease resistance, their down-regulation suggest that *P*. *manihoti* feeding may have suppressed the activation of their immune response in cassava.

Most of the pathogenesis-related proteins in P40/1 were down-regulated except two HSP20-like chaperones superfamily proteins (Manes.02G124800, Manes.02G124700), which were highly up-regulated in mealybug infested tissues harvested at 24 hpi compared with mealybug infested tissues at 72 hpi (S3 Table). These two HSP20-like chaperones superfamily proteins were different from those that were up-regulated in AR23.1 above. We also observed up-regulation of a WRKY DNA-binding protein 27 (Manes.02G017100) when mealybug infested tissues harvested at 24 hpi were compared with infested tissues at 72 hpi. This observation suggested a later (after 24 hours) induction of the two HSP20-like chaperones superfamily proteins (Manes.02G124800, Manes.02G124700) and WRKY DNA-binding protein 27 (Manes.02G017100).

We further observed the up-regulation of UDP-glucosyl transferase 74B1 (Manes.01G196500) when tissues harvested at 24 hours were compared with those harvested at 72 hours of mealybug infestation, suggesting a later induction of the gene during insect attack. The same gene was found to be up-regulated when mealybug infested tissues harvested at 24 hpi were compared with non-infested mock tissues at the same time further confirming a theory of late induction of the gene. UDP- glucosyl transferases have been reported to be responsible for the last step in the biosynthesis of linamarin and lotaustralin, which are the major cyanogenic glucosides in cassava [55].

KO terms could be assigned to 52 out of the 206 (25.2%) DEGs identified, and were subsequently mapped to 12 KEGG pathways (Fig 7B). DE genes that were mapped to the plant-pathogen interactions pathway were down-regulated (Fig 7B) while those that were mapped to the plant hormone signal transduction pathways were both up- (MAP kinase substrate 1, Auxin efflux carrier 17 family protein, Jamonate-zim-domain protein 8, Highly ABA-393 induced PP2C gene 3) and down-regulated [(pathogenesis-related thaumatin superfamily protein, Pathogenesis-related 4) (Fig 7B)]. It is important to note that due to lack of representative KO terms, not all genes were mapped and therefore the output from KEGG analysis would not be representative of all the DEGs.

### Comparison of expression patterns between AR23.1 and P40/1

Gene expression was further compared between AR23.1 and P40/1 to determine whether there were any genotype-specific expression patterns. Within the GO functional classification, genes under secondary metabolic process category were significantly enriched in AR23.1 in comparison with P40/1 (Fig 6). On the other hand, genes under regulation of molecular function, cellular component biogenesis and enzyme regulator categories were more significantly enriched in P40/1 than in AR23.1 (Fig 6). Under the metabolic pathway analysis using the KEGG database, genes under ABC transporters and MAPK signalling pathways, both of which have been reported to be involved in plant defence, were only present in AR23.1 and not in P40/1 (Fig 7). There were both down- and up-regulated genes that were mapped to the plant-pathogen interactions and plant hormone signal transduction pathway (Table 7) in genotype AR23.1, while in P40/1, there were no up-regulated genes classified under plant-pathogen interactions pathway (Table 7). It must be noted that the genes shown in Table 7 are an under-representation of all plant-pathogen interactions and plant-hormone signal transduction genes that were found to be differentially expressed in both AR23.1 and P40/1 due to lack of representative KO terms.

**Table 7:** Differentially expressed genes in AR23.1 and P40/1 that were mapped to plant-pathogen interactions and plant hormone signal transduction KEGG pathways.

**AR23.1—Plant pathogen interaction**			**P40/1—Plant pathogen interaction**	
**Up-regulated**	**Gene ID**	**Gene annotation**	**Gene ID**	**Gene annotation**
	Manes.01G147500	Myb domain protein 4		
	Manes.02G075400	Calmodulin like 23	N/A	
	Manes.01G235800	Myb domain protein 55		
	Manes.02G051000	Cyclic nucleotide-regulated ion channel family protein		
**Down-regulated**	Manes.01G142200	Leucine-rich repeat receptor-like protein kinase family protein	Manes.01G241000	3-ketoacyl-CoA synthase 1
	Manes.02G124600	HSP20-like chaperones superfamily protein	Manes.01G119000	HSP20-like chaperones superfamily protein
	Manes.01G252200	WRKY DNA-binding protein 56	Manes.01G042200	Heat shock protein 21
	Manes.01G235800	Myb domain protein 55	Manes.01G116700	Calmodulin like 23
	Manes.02G125200	HSP20-like chaperones superfamily protein	Manes.02G017100	WRKY DNA-binding protein 27
	Manes.01G119000	HSP20-like chaperones superfamily protein	Manes.02G124700	HSP20-like chaperones superfamily protein
	Manes.02G124700	HSP20-like chaperones superfamily protein	Manes.01G203500	Calmodulin-like 11
	Manes.01G189000	WRKY DNA-binding protein 72		
	Manes.01G273700	WRKY family transcription factor		
	Manes.01G203500	Calmodulin-like 11		
**AR23.1—Plant hormone signal transduction**			**P40/1—Plant hormone signal transduction**	
**Up-regulated**	**Gene ID**	**Gene annotation**	**Gene ID**	**Gene annotation**
	Manes.02G099700	MAP kinase substrate 1	Manes.02G176600	MAP kinase substrate 1
	Manes.01G085400	ethylene responsive element binding factor 1	Manes.02G115200	Auxin efflux carrier family protein
	Manes.02G115200	Auxin efflux carrier family protein		
	Manes.01G050400	Pathogenesis-related thaumatin superfamily protein		
**Down-regulated**	Manes.02G105300	Pathogenesis-related thaumatin superfamily protein	Manes.02G105400	Pathogenesis-related thaumatin superfamily protein
			Manes.02G105300	Pathogenesis-related thaumatin superfamily protein

Four genes [Gibberellin 2-oxidase 6 (Manes.01G231100), HSP20-like chaperones superfamily protein (Manes.02G124600), alpha/beta-Hydrolases superfamily protein (Manes.01G274500), and GDSL-like Lipase/Acylhydrolase superfamily protein (Manes.02G189200),] that were up-regulated in AR23.1 were down-regulated in P40/1 at 24 hours of mealybug infestation. A “pectin lyase-like superfamily” protein (Manes.02G092000) that was up-regulated (+1.73) in P40/1 at 72 hours of mealybug infestation was down-regulated in AR23.1 (-1.51) although a different “pectin lyase-like superfamily” protein (Manes.01G231200) was up-regulated in AR23.1 at the same time period (S2 and S3 Tables). We also observed the up-regulation of a “Calmodulin like 23” (Manes.02G075400) in AR23.1 and a down-regulation of the same family protein [“Calmodulin like 23” (Manes.01G116700)] in P40/1 (Table 6). The UDP-glucosyl transferase (Manes.02G054300) was also down-regulated in AR23.1, whereas in P40/1, UDP-glucosyl transferase 74B1” (Manes.01G196500) was up-regulated.

### Validation of expressed transcripts by RT-qPCR

**Fig 8:**
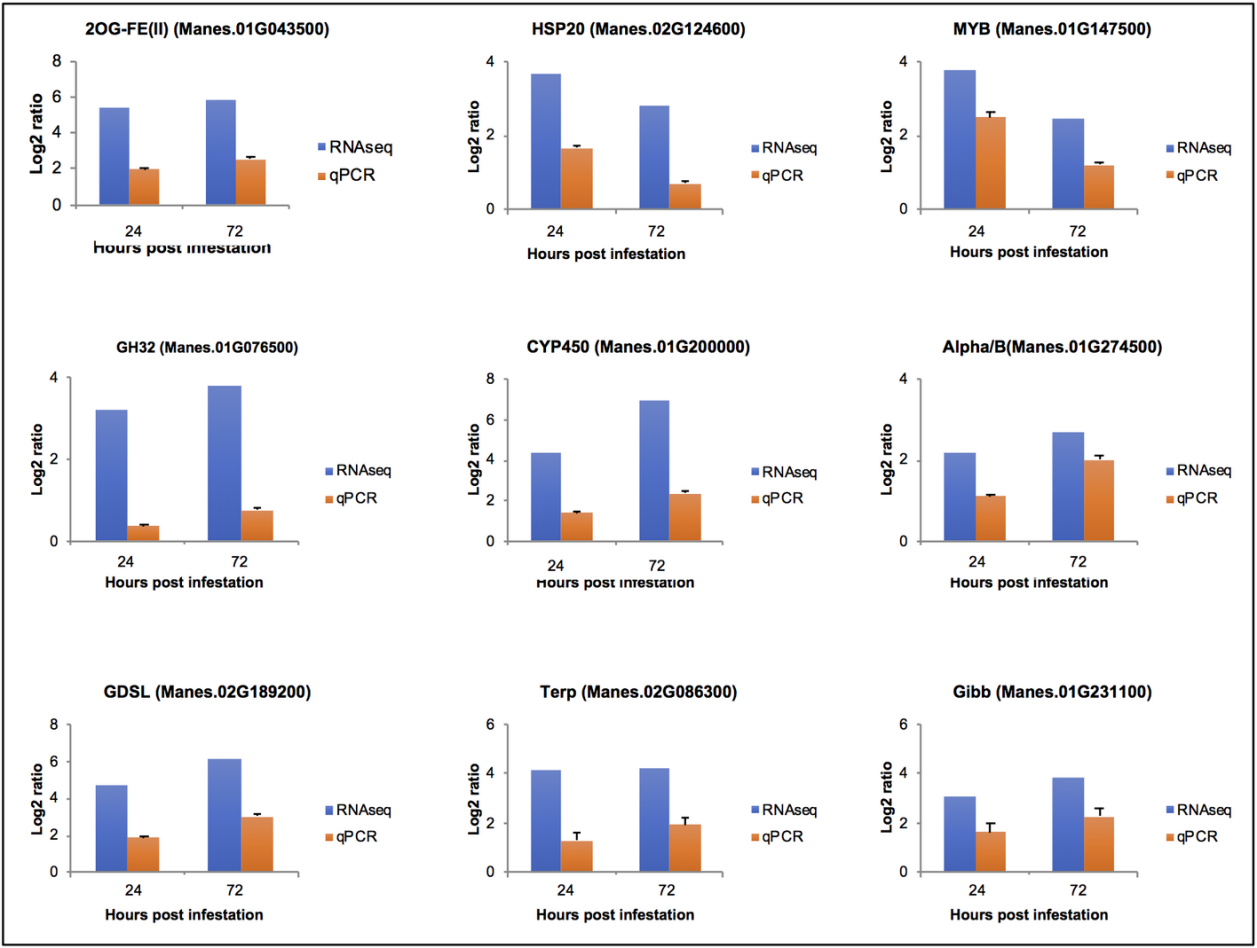
Comparative expression levels by RNA-seq and real-time RT-qPCR of nine cassava up-regulated genes in genotype AR23.1 in response to mealybug infestation at 24 and 72 hours calculated with reference to GTPb. Error bars on each qPCR column indicate the standard error (SE) from three biological replicates.

The expression of nine candidate mealybug induced defence-related genes (Table 3) was validated using qRT-PCR, with GTPb as reference gene. BestKeeper proved that GTPb was the most stable reference gene for this analysis (S4 and S5 Figs). The nine DEGs were selected based on their likely defence role in response to herbivore infestation as reported in other plant systems [8, 31, 47, 56]. The analysis of the data provided the log2 ratios between time points in relation to reference gene GTPb (Fig 8). Consistent with RNA-seq data, 2OG-FE(II) gene was among the most highly expressed at 24 hours and 72 hours of mealybug infestation, followed by CYP450, GH32 and Alpha/B family genes with reference to GTPB (Fig 8). Although the two procedures (RNA-seq and qPCR) are very different, they could independently confirm the general trend of expression of the selected genes upon mealybug infestation (Fig 8).

## Discussion

Previous cassava mealybug studies in South Africa have relied on morphological identification methods, which are often laborious and dependent on the female adult [15]. In this study, the identification of cassava mealybug species was confirmed as *P*. *manihoti* using both morphological and molecular techniques in two South African provinces, KwaZulu-Natal and Mpumalanga. Important to note is that these two regions are the main cassava producing areas in this country. Therefore, the current study serves as a baseline to conduct a more thorough mealybug sampling strategy in KwaZulu-Natal and Mpumalanga, as well as in all cassava growing regions in South Africa and the rest of the African continent.

The contrasting differences observed in the two cassava genotypes for resistance to mealybug indicate the possibility of using genetics to develop more tolerant cassava varieties in the region. Tolerance/resistance in genotype AR23.1 was hallmarked by minimum colonization by *P*. *manihoti* and a relatively long-term interaction (35 dpi) with mealybugs without showing severe symptoms of infestation/feeding. A similar observation has been reported in soybean (*Glycine max*) [57] with soybean aphids (*Aphis glycine*) at 28 days after infestation, as well as in tomato (*Solanum lycopersicum*) plants infested with mealybug *Phenacoccus solenopsis* [58]. Although there have been many reports suggesting the production of toxins such as linamarin and lotaustralin as a likely mechanism of defence to insects in cassava [55], our study did not observe any evidence for such a mechanism. We rather observed down-regulation (-1.36 log2 fold) of UDP-glucosyl transferase (Manes.02G054300) in the resistant genotype AR23.1. This further suggests that resistance-related genes and not the production of toxins such as linamarin and lotaustralin largely conferred the defence observed in AR23.1. UDP- glucosyl transferases have been reported to be responsible for the last step in the biosynthesis of linamarin and lotaustralin, which are the major cyanogenic glucosides in cassava [55]. In the same genotype, we observed massive up-regulation of pathogenesis-related (PR) proteins within 24 hours of mealybug infestation in comparison with the susceptible genotype. This suggests the involvement of PR proteins including major R genes such as the two Leucine-rich repeat receptor-like protein kinase (LRR-PK) that were up-regulated within the first 24 hours of mealybug infestation. These two R genes were not up-regulated in the susceptible genotype P40/1. These results are consistent with those of similar studies done in *Arabidopsis thaliana* [30], soybean (*Glycine max*) [59], *Medicago truncatula* [60], *Brassica oleracea* [61] and grapevines (*Vitis vinifera*) [31].

Even though more detailed studies will be required to establish the true mechanism of tolerance/resistance observed in AR23.1, we were able to detect the induction of specific leucine-rich repeat (LRR) TIR-NBS-LRR and LRR receptor-like protein kinase (PK) resistance family proteins [62] in this study. TIR-NBS-LRR are involved in the division of signalling pathways leading to disease resistance [63, 64], while LRR-PK resistance family proteins are involved in regulation of developmental and defence-related processes [65]. These R proteins were up-regulated in AR23.1 and were either absent or down-regulated in P40/1 (S2 and S3 Tables), suggesting that the R proteins may be involved in recognizing the presence of the insect. Other defence-associated genes such as MYB domain transcription factors, HSP20-like chaperone superfamily proteins and calmodulin like proteins were also differentially expressed in AR23.1 and not in P40/1. Many of these induced defence-related proteins have also been observed in several other plant-insect and plant-pathogen interaction studies [8, 27, 29, 31, 47, 66, 67]. We did not observe consistencies in the expression pattern of some of the stress-related proteins such as Cytochrome P450 family protein. Cytochrome P450s are involved in the metabolism of plant allelochemicals [68] which are induced as a result of abiotic and biotic stresses [28, 29, 47, 68, 69].

Although transcriptional changes have been observed as early as 1 hpi in other studies with different pests including diamondback moth (*Plutella xylostella*) in bittercress (*Barbarea vulgaris*) [47, 70] and as late as 96 hpi in cotton [31] and 120 hpi in tomato [58], we relied on previous successful transcriptomic studies [8, 31, 56, 58], majority of which settled on sampling at 24 and 72 hpi. A higher number (50 and 42 for AR23.1 and P40/1, respectively) of down-regulated genes at 24 hours of mealybug infestation compared with up-regulated DEGs (42 and 25 in AR23.1 and P40/1, respectively) in both genotypes was observed (Table 4), demonstrating initial suppression of certain classes of genes in response to mealybug feeding. Other studies have reported similar results in *Arabidopsis thaliana* [8], tomato [56] and *Barbarea vulgaris* [47], where initial suppression of genes in response to pathogen attack was observed. The suppression of genes may be a pest survival mechanism and has been reported in the interaction between sap-sucking insects (*Bemisia tabacci* and *Aphis gossypii*) with cotton (*Gossypium hirsutum*) [71].

In the present study, important evidence was also provided through the GO analysis of specific processes involved in transcriptome changes in cassava leaves in response to *P*. *manihoti* feeding. Gene ontology (GO) annotation of DEGs expressed in response to mealybug feeding revealed differential responses to stress, regulation of biological processes, cell and binding categories between the resistant and susceptible genotypes (Fig 6). The enrichment of the same GO categories in response to biotic and abiotic stress is consistent with other studies [28, 47].

Insect feeding has been shown to result in both constitutive and induced defence mechanisms, which lead to activation of signal cascades and release of volatiles for protection [72]. The genotype AR23.1 showed a high level of enrichment for molecular and signal transducers under GO term classification (Fig 6). The role of signal transducers such as jasmonates in defence against insects has been extensively reported [8, 23, 29, 73]. The enrichment of signal transducers in AR23.1 and absence of the same class in P40/1 (Table 6; S2 and S3 Tables) strongly suggests a direct role in defence mechanisms in AR23.1 against cassava mealybug. The specific up-regulation of signal transducers in AR23.1 (Table 6; S2 and S3 Tables) and absence of the same in P40/1 further supports this hypothesis.

In this study, gibberellin 2-oxidase 6 (Manes.01G231100), HSP20-like chaperones superfamily protein (Manes.02G124600), alpha/beta-Hydrolases superfamily protein (Manes.01G274500) and GDSL-like Lipase/acylhydrolase superfamily protein (Manes.02G189200) were up-regulated in AR23.1 but down-regulated in P40/1 at 24 hours of mealybug infestation (S2 and S3 Tables), implicating these genes in a biotic stress [60, 67, 74–79] response to *P*. *manihoti* infestation. Gibberellin 2-oxidase family proteins are involved in plant development by catalysing the 2-beta-hydroxylation of biologically active gibberellins [80, 81], while HSP20-like chaperones superfamily protein act as molecular chaperones by assisting in the refolding of partially denatured proteins [82]. Alpha/beta-hydrolase superfamily protein function as hydrolases, hormone precursors or transporters and chaperones of other proteins [83], while the GDSL-like lipase/acylhydrolase superfamily proteins play a role in biotic stress response by breaking down fatty acids, lipids and isoprenoids in plants [84]. These genes have also been associated with both abiotic and biotic stress responses in various crops including *Arabidopsis* [66, 85], cotton [76], soybean [74], rice [77, 78, 84] chickpea [86] and cassava [67].

Our results also show a high level of induction of 2-oxogluterate (2OG) and Fe (II)-dependent oxygenase superfamily proteins and MYB domain transcription factor in AR23.1 and not in P40/1. The 2-oxogluterate (2OG) and Fe (II)-dependent oxygenase superfamily proteins have been reported to be involved in plant defence [87] by catalysing the formation of plant hormones while the MYB domain family proteins are involved in regulation of transcription factors in plants [88, 89]. Previous studies have demonstrated up-regulation of these afore-mentioned family genes in response to herbivore infestation [31, 56, 66, 73], suggesting that these are associated with insect feeding response. For instance, Coppola et al. [56] and Artico et al. [29] reported up-regulation of genes such as MYB domain transcription factor and 2-oxogluterate (2OG) and Fe (II)-dependent oxygenase superfamily protein post-infestation by *Macrosiphum euphorbiae* in tomato and *Anthonomus grandis* in cotton, respectively. There are also similar reports by Timm and Reinecke [31] in response to *Phenacoccus ficus* infestation in vines.

It was also interesting to note that some members of the same gene families were both induced and supressed at the same time-points in the same genotype. For example, heat shock proteins, gibberellin-regulated proteins and Cytochrome P450 family proteins were both up- and down-regulated at 24 and 72 hours of mealybug infestation compared to non-infested mock tissues in the partially resistant AR23.1 genotype (S2 Table). A similar trend was observed in a study by Broekgaarden et al. [73], in which expressed proteins (unknown gene proteins) were reported to be induced or repressed in cabbage Rivera and Christmas Drumhead cultivars post infestation by cabbage aphid *Brevicoryne brassicae*. These results point to diversity in the functions of the same gene family in response to herbivorous insects’ feeding.

Although we observed a significant number of DEGs, most of them were not assigned specific KEGG Orthology (KO) terms due to the incomplete annotation of the cassava genome, making it difficult to use the KEGG database and establish detailed pathway analyses. Nevertheless, the DEGs that were assigned KO terms in the present study demonstrated an abundance of genes playing a role in plant hormone signal transduction and plant-pathogen interactions. The plant hormone signal transduction pathways rely on signal molecules such as abscisic acid ABA, JA, ET, and SA which are elicited in response to insect interactions [8]. Mealybugs appeared to elicit a defence reaction based on the cross-communication of different hormone-related signalling pathways. *P*. *manihoti* feeding suppressed more defence genes compared with induced genes in both the susceptible P40/1 and partially resistant AR23.1 cassava genotypes. Many genes involved in JA, SA, ET and GA (Table 6; S2 and S3 Tables) biosynthesis were down-regulated post infestation by mealybugs at different time-points. The suppression of JA-regulated genes and defence metabolites in tomato plants infested with *Phenacoccus solenopsis* was also reported in a recent study by Zhang et al. [58], demonstrating the intervention of JA pathway in defence response to *Phenacoccus solenopsis* feeding. Several studies in the past have confirmed the induction of signal transduction pathways and primary induced defence responses upon pest infestation in different plants including *Arabidopsis* [66], tomato [56], soybean [59] and *Medicago truncatula* [90]. In addition to PAMP-triggered immunity (PTI), effector-triggered immunity (ETI), represented by differentially expressed transcripts “calcium binding” and “calmodulin proteins”, “heat shock” proteins and transcription factors such as those of the MAPK and WRKY families among others (S2 and S3 Tables) were observed in both AR23.1 and P40/1 genotypes. ETI is also implicated, from other transcriptome studies, in herbivore infestation and pathogen infection [29, 56, 66, 91–94] as well as in other abiotic stress-related studies such as drought, cold stress and heat treatments [28, 69]. Collectively, this and other studies suggest that the response to insect feeding or damage in plants could be highly conserved across different plant hosts and different insect species, and that commonalities in molecular responses with other abiotic stresses also occurs.

The KEGG database revealed the enrichment of ABC transporters and MAPK signalling pathways specifically in AR23.1 and not P40/1. ABC transporters play a role in organ growth, plant nutrition, plant development and in response to abiotic and biotic stress [95], while MAPKs play a role in signalling of a variety of abiotic and biotic stress [96], and elicitation of transporters and signalling molecules in AR23.1 suggest an induced defence response to mealybug feeding not observed in the susceptible genotype P40/1.

In conclusion, transcript profiling of AR23.1 and P40/1 interaction with *P*. *manihoti* in the present study revealed that mealybug feeding induces transcriptome reprogramming associated with basal defence and abiotic stress hallmarks in cassava plants at a very early stage of infestation. However, the link between these early hormone signalling responses and downstream biochemical responses, and tolerance/resistance to mealybug feeding, needs to be established. In cassava, fewer transcriptome studies have been published in response to abiotic and biotic stresses [27, 67, 97–103]. Reports demonstrating the involvement of secondary metabolites in deterring insect feeding [104, 105] will require a metabolomics study to resolve this mechanism. Our current results provide strong evidence for the induction of plant resistance genes, hormonal signal transduction and basal immunity-related compounds, in response to mealybug feeding. These results will also form a basis for more detailed future studies on the specific role of some of the differentially expressed genes identified.

## Supporting information

S1 Fig Schematic representation of the experimental approach for RNA isolation and cDNA preparation from cassava genotypes infested with mealybugs. Three independent biological replicates were performed.
(PDF)

S2 Fig Correlations of AR23.1 leaf samples infested with mealybugs (24 and 72 hours post infestation) compared to mock (non-infested). (PDF)

S3 Fig Correlations of P40/1 leaf samples infested with mealybugs (24 and 72 hours post infestation) compared to mock (non-infested). (PDF)

S4 Fig Descriptive statistics of four candidate housekeeping genes (HKG) based on their crossing point (CP) values. (PDF)

S5 Fig Expression stability of the four candidate housekeeping genes (UBQ10, GTPb, EF1 and Actin) in cassava leaves. (PDF)

S1 Table A list of raw and trimmed reads generated by Illumina HiSeq 2500 per time point after pooling across the three biological replicates of AR23.1 (resistant) and P40/1 (susceptible) per genotype; and subsequently mapping to the cassava reference genome (*Mesculenta*_305_v6.1). (PDF)

S2 Table List of AR23.1 differentially expressed genes in cassava leaves in response to mealybug infestation at 24 and 72 hours post infestation. (PDF)

S3 Table List of P40/1 differentially expressed genes in cassava leaves in response to mealybug infestation at 24 and 72 hours post infestation. (PDF)

S4 Table List of Gene Ontology (GO) terms for AR23.1 and P40/1 cassava genotypes following mealybug infestation categorized into Biological process, Molecular function and Cellular component. (PDF)

## References

[1] Beltrán J, Prías M, Al-Babili S, Ladino Y, López D, Beyer P, et al Expression pattern conferred by a glutamic acid-rich protein gene promoter in field-grown transgenic cassava (Manihot esculenta Crantz). Planta. 2010;231(6):1413–24. 10.1007/s00425-010-1144-7 20336312

[2] Cock JH. Cassava: a basic energy source in the tropics. Science. 1982;218(4574):755–62. 7134971 10.1126/science.7134971

[3] El-Sharkawy MA. Cassava biology and physiology. Plant Molecular Biology. 2003;53(5):621–41. 10.1023/B:PLAN.0000019065.31490.06 15669146

[4] Fregene M, Okogbenin E, Mba C, Angel F, Suarez MC, Janneth G, et al Genome mapping in cassava improvement: Challenges, achievements and opportunities. Euphytica. 2001;120(1):159–65.

[5] FAOSTAT. Cassava production trends in Africa, 2005–2014 www.faostat3.fao.org/browse/rankings/commodities_by_regions/E 2016.

[6] Nweke F, Spencer D, Lynam J. The cassava transformation. Africa’s best kept secret Michigan State University, East Lansing. 2002:203–14.

[7] Beeching JR, Han Y, Gomez-Vasquez R, Day RC, Cooper RM. Wound and defense responses in cassava as related to post-harvest physiological deterioration. 1998 p. 231–48.

[8] De Vos M, Van Oosten VR, Van Poecke RM, Van Pelt JA, Pozo MJ, Mueller MJ, et al Signal signature and transcriptome changes of Arabidopsis during pathogen and insect attack. Molecular Plant-Microbe Interactions. 2005;18(9):923–37. 10.1094/MPMI-18-0923 16167763

[9] Dixon A, Asiedu R, Hahn S. Cassava germplasm enhancement at the International Institute of Tropical Agriculture (IITA). In: Akoroda, MO and Arene, OB (eds) Proceedings of the 4th ISTRC-AB Symposium, IITA, Nigeria. 1992:83–7.

[10] Hillocks RJ, Wydra K. Bacterial, fungal and nematode diseases In: Hillocks RJ, Thresh JM and Bellotti AC (eds) Cassava: Biology, Production and Utilization, CABI Publishing, CIAT, Cali, Colombia and University of Greenwich, Kent, UK 2002:261–80.

[11] Lin F, Zhao M, Baumann DD, Ping J, Sun L, Liu Y, et al Molecular response to the pathogen Phytophthora sojae among ten soybean near isogenic lines revealed by comparative transcriptomics. BMC genomics. 2014;15(1):18–30. 24410936 10.1186/1471-2164-15-18 PMC3893405

[12] Mahungu N, Dixon AG, Kumbira J. Breeding cassava for multiple pest resistance in Africa. African Crop Science Journal. 2009;2:539–52.

[13] Ceballos H, Iglesias CA, Pérez JC, Dixon AG. Cassava breeding: opportunities and challenges. Plant Molecular Biology. 2004;56(4):503–16. 10.1007/s11103-004-5010-5 15630615

[14] Hillocks RJ, Thresh J, Bellotti A. Cassava: biology, production and utilization: CABI; 2002.

[15] Millar I. Mealybug genera (Hemiptera: Pseudococcidae) of South Africa: identification and review. African Entomology. 2002;10(2):185–233.

[16] Sethusa M, Millar I, Yessoufou K, Jacobs A, Van der Bank M, Van der Bank H. DNA barcode efficacy for the identification of economically important scale insects (Hemiptera: Coccoidea) in South Africa. African Entomology. 2014;22(2):257–66.

[17] Herren HR, Neuenschwander P. Biological control of cassava pests in Africa. Annual Review of Entomology. 1991;36(1):257–83.

[18] Bertschy C, Turlings TC, Bellotti AC, Dorn S. Chemically-mediated attraction of three parasitoid species to mealybug-infested cassava leaves. Florida Entomologist. 1997:383–95.

[19] Calatayud P-A, Le Rü BP. Cassava-mealybug interactions (eds) IIDR, editor. CIAT, Cali, Colombia: In: Institut De Recherche (eds); 2006.

[20] Le Ru B, Calatayud P-A. Interactions between cassava and arthropod pests. 2009.

[21] Bari R, Jones JD. Role of plant hormones in plant defence responses. Plant Molecular Biology. 2009;69(4):473–88. 10.1007/s11103-008-9435-0 19083153

[22] Wu J, Baldwin IT. New insights into plant responses to the attack from insect herbivores. Annual Review of Genetics. 2010;44:1–24. 10.1146/annurev-genet-102209-163500 20649414

[23] Huang X-Z, Chen J-Y, Xiao H-J, Xiao Y-T, Wu J, Wu J-X, et al Dynamic transcriptome analysis and volatile profiling of Gossypium hirsutum in response to the cotton bollworm Helicoverpa armigera. Scientific Reports. 2015;5. 10.1038/srep11867 PMC4493570 26148847

[24] Wang Y, Wu W-H. Potassium transport and signaling in higher plants. Annual Review of Plant Biology. 2013;64:451–76. 10.1146/annurev-arplant-050312-120153 23330792

[25] Moran PJ, Thompson GA. Molecular responses to aphid feeding in Arabidopsis in relation to plant defense pathways. Plant Physiology. 2001;125(2):1074–85. 11161062 10.1104/pp.125.2.1074 PMC64906

[26] Lorenzo O, Solano R. Molecular players regulating the jasmonate signalling network. Current Opinion in Plant Biology. 2005;8(5):532–40. 10.1016/j.pbi.2005.07.003 16039901

[27] Allie F, Pierce EJ, Okoniewski MJ, Rey C. Transcriptional analysis of South African cassava mosaic virus-infected susceptible and tolerant landraces of cassava highlights differences in resistance, basal defense and cell wall associated genes during infection. BMC genomics. 2014;15(1):1006–35. 25412561 10.1186/1471-2164-15-1006 PMC4253015

[28] An D, Yang J, Zhang P. Transcriptome profiling of low temperature-treated cassava apical shoots showed dynamic responses of tropical plant to cold stress. BMC genomics. 2012;13(1):64–87. 22321773 10.1186/1471-2164-13-64 PMC3339519

[29] Artico S, Ribeiro-Alves M, Oliveira-Neto OB, de Macedo LL, Silveira S, Grossi-de-Sa MF, et al Transcriptome analysis of Gossypium hirsutum flower buds infested by cotton boll weevil (Anthonomus grandis) larvae. BMC genomics. 2014;15(1):854–77. 25280771 10.1186/1471-2164-15-854 PMC4234063

[30] Ehlting J, Chowrira SG, Mattheus N, Aeschliman DS, Arimura G-I, Bohlmann J. Comparative transcriptome analysis of Arabidopsis thaliana infested by diamond back moth (Plutella xylostella) larvae reveals signatures of stress response, secondary metabolism, and signalling. BMC genomics. 2008;9(1):154–173. 18400103 10.1186/1471-2164-9-154 PMC2375910

[31] Timm AE, Reineke A. First insights into grapevine transcriptional responses as a result of vine mealybug Planococcus ficus feeding. Arthropod-Plant Interactions. 2014;8(6):495–505.

[32] Belhaj K, Chaparro-Garcia A, Kamoun S, Nekrasov V. Plant genome editing made easy: targeted mutagenesis in model and crop plants using the CRISPR/Cas system. Plant methods. 2013;9(1):1–10. 10.1186/1746-4811-9-1 24112467 PMC3852272

[33] Williams DJ, de Willink MCG. Mealybugs of central and South America: CAB International, Wallingford, UK; 1992 pp 635

[34] Dowton M, Austin A. Phylogenetic relationships among the microgastroid wasps (Hymenoptera: Braconidae): combined analysis of 16S and 28S rDNA genes and morphological data. Molecular Phylogenetics and Evolution. 1998;10(3):354–66. 10.1006/mpev.1998.0533 10051388

[35] Whiting MF, Carpenter JC, Wheeler QD, Wheeler WC. The Strepsiptera problem: phylogeny of the holometabolous insect orders inferred from 18S and 28S ribosomal DNA sequences and morphology. Systematic Biology. 1997;46(1):1–68. 11975347 10.1093/sysbio/46.1.1

[36] Tautz D, Hancock JM, Webb DA, Tautz C, Dover GA. Complete sequences of the rRNA genes of Drosophila melanogaster. Molecular Biology and Evolution. 1988;5(4):366–76. 10.1093/oxfordjournals.molbev.a040500 3136294

[37] von Dohlen CD, Moran NA. Molecular phylogeny of the Homoptera: a paraphyletic taxon. Journal of Molecular Evolution. 1995;41(2):211–23. 7666451 10.1007/BF00170675

[38] Staden R, Judge DP, Bonfield JK. Sequence assembly and finishing methods In: Andreas D. Baxevanis aBFFOe, editor. New York, USA: New York, USA: John Wiley & Sons; 1998.

[39] Murashige T, Skoog F. A revised medium for rapid growth and bio assays with tobacco tissue cultures. Physiologia Plantarum. 1962;15(3):473–97.

[40] Zhang X, Zou F, Wang W, editors. Fastanova: an efficient algorithm for genome-wide association study. Proceedings of the 14th ACM SIGKDD international conference on Knowledge discovery and data mining; 2008: ACM. PMC2951741 20945829

[41] Nweke F, Otim-Nape G, Dixon A, Asadu C, Bua A, Ajobo O, et al. Production prospects for cassava in Uganda. Collaborative Study of Cassava in Africa (COSCA). Collaborative Study of Cassava in Africa (COSCA): Working paper No. 17, International Institute of Tropical Agriculture, Ibadan, Nigeria.; 1999.

[42] Bianchi V, Colantoni A, Calderone A, Ausiello G, Ferre F, Helmer-Citterich M. DBATE: database of alternative transcripts expression. Database. 2013;2013:50–59. 10.1093/database/bat050 PMC5654372 23842462

[43] Bolger AM, Lohse M, Usadel B. Trimmomatic: a flexible trimmer for Illumina sequence data. Bioinformatics. 2014;30:2114–20. 10.1093/bioinformatics/btu170 24695404 PMC4103590

[44] Kim D, Pertea G, Trapnell C, Pimentel H, Kelley R, Salzberg SL. TopHat2: accurate alignment of transcriptomes in the presence of insertions, deletions and gene fusions. Genome Biology. 2013;14(4):36–48. 10.1186/gb-2013-14-4-r36 PMC4053844 23618408

[45] Trapnell C, Roberts A, Goff L, Pertea G, Kim D, Kelley DR, et al Differential gene and transcript expression analysis of RNA-seq experiments with TopHat and Cufflinks. Nature protocols. 2012;7(3):562–78. 10.1038/nprot.2012.016 22383036 PMC3334321

[46] Park S-J, Huang Y, Ayoubi P. Identification of expression profiles of sorghum genes in response to greenbug phloem-feeding using cDNA subtraction and microarray analysis. Planta. 2006;223(5):932–47. 10.1007/s00425-005-0148-1 16292568

[47] Wei X, Zhang X, Shen D, Wang H, Wu Q, Lu P, et al Transcriptome analysis of Barbarea vulgaris infested with diamondback moth (Plutella xylostella) larvae. PLoS One. 2013;8:64481–99. 10.1371/journal.pone.0064481 PMC3655962 23696897

[48] Ye J, Fang L, Zheng H, Zhang Y, Chen J, Zhang Z, et al WEGO: a web tool for plotting GO annotations. Nucleic Acids Research. 2006;34(2):293–7. 10.1093/nar/gkl031 PMC1538768 16845012

[49] Kanehisa M, Goto S, Kawashima S, Okuno Y, Hattori M. The KEGG resource for deciphering the genome. Nucleic Acids Research. 2004;32(1):277–80. 10.1093/nar/gkh063 PMC308797 14681412

[50] Moriya Y, Itoh M, Okuda S, Yoshizawa AC, Kanehisa M. KAAS: an automatic genome annotation and pathway reconstruction server. Nucleic Acids Research. 2007;35:182–5. 10.1093/nar/gkm321 PMC1933193 17526522

[51] Moreno I, Gruissem W, Vanderschuren H. Reference genes for reliable potyvirus quantitation in cassava and analysis of Cassava brown streak virus load in host varieties. Journal of Virological Methods. 2011;177(1):49–54. 10.1016/j.jviromet.2011.06.013 21756941

[52] Livak KJ, Schmittgen TD. Analysis of relative gene expression data using real-time quantitative PCR and the 2− ΔΔCT method. Methods. 2001;25(4):402–8. 10.1006/meth.2001.1262 11846609

[53] Pfaffl MW. Quantification strategies in real-time PCR. AZ of Quantitative PCR. 2004;1:89–113.

[54] Pfaffl MW, Tichopad A, Prgomet C, Neuvians TP. Determination of stable housekeeping genes, differentially regulated target genes and sample integrity: BestKeeper–Excel-based tool using pair-wise correlations. Biotechnology Letters. 2004;26:509–15. 15127793 10.1023/b:bile.0000019559.84305.47

[55] Jørgensen K, Morant AV, Morant M, Jensen NB, Olsen CE, Kannangara R, et al Biosynthesis of the cyanogenic glucosides linamarin and lotaustralin in cassava: isolation, biochemical characterization, and expression pattern of CYP71E7, the oxime-metabolizing cytochrome P450 enzyme. Plant Physiology. 2011;155(1):282–92. 10.1104/pp.110.164053 21045121 PMC3075754

[56] Coppola V, Coppola M, Rocco M, Digilio MC, D’Ambrosio C, Renzone G, et al Transcriptomic and proteomic analysis of a compatible tomato-aphid interaction reveals a predominant salicylic acid-dependent plant response. BMC genomics. 2013;14(1):515–32. 23895395 10.1186/1471-2164-14-515 PMC3733717

[57] Mian M, Hammond RB, St Martin SK. New plant introductions with resistance to the soybean aphid. Crop Science. 2008;48(3):1055–61.

[58] Zhang P-J, Huang F, Zhang J-M, Wei J-N, Lu Y-B. The mealybug Phenacoccus solenopsis suppresses plant defense responses by manipulating JA-SA crosstalk. Scientific Reports. 2015;5:9354–60. 10.1038/srep09354 25790868 PMC4366759

[59] Li Y, Zou J, Li M, Bilgin DD, Vodkin LO, Hartman GL, et al Soybean defense responses to the soybean aphid. New Phytologist. 2008;179(1):185–95. 10.1111/j.1469-8137.2008.02443.x 18422900

[60] Gao LL, Kamphuis LG, Kakar K, Edwards OR, Udvardi MK, Singh KB. Identification of potential early regulators of aphid resistance in Medicago truncatula via transcription factor expression profiling. New Phytologist. 2010;186(4):980–94. 10.1111/j.1469-8137.2010.03229.x 20345634

[61] Broekgaarden C, Poelman EH, Steenhuis G, Voorrips RE, Dicke M, Vosman B. Genotypic variation in genome-wide transcription profiles induced by insect feeding: Brassica oleracea–Pieris rapae interactions. BMC genomics. 2007;8(1):239–51. 17640338 10.1186/1471-2164-8-239 PMC1940009

[62] Gururani MA, Venkatesh J, Upadhyaya CP, Nookaraju A, Pandey SK, Park SW. Plant disease resistance genes: current status and future directions. Physiological and Molecular Plant Pathology. 2012;78:51–65.

[63] Century KS, Shapiro AD, Repetti PP, Dahlbeck D, Holub E, Staskawicz BJ. NDR1, a pathogen-induced component required for Arabidopsis disease resistance. Science. 1997;278(5345):1963–5. 9395402 10.1126/science.278.5345.1963

[64] Parker JE, Holub EB, Frost LN, Falk A, Gunn ND, Daniels MJ. Characterization of eds1, a mutation in Arabidopsis suppressing resistance to Peronospora parasitica specified by several different RPP genes. The Plant Cell. 1996;8(11):2033–46. 10.1105/tpc.8.11.2033 8953768 PMC161332

[65] Torii KU. Leucine-rich repeat receptor kinases in plants: structure, function, and signal transduction pathways. International review of cytology. 2004;234:1–46. 10.1016/S0074-7696(04)34001-5 15066372

[66] Bricchi I, Bertea CM, Occhipinti A, Paponov IA, Maffei ME. Dynamics of membrane potential variation and gene expression induced by Spodoptera littoralis, Myzus persicae, and Pseudomonas syringae in Arabidopsis. PLoS One. 2012;7:46673–92. 10.1371/journal.pone.0046673 PMC3484130 23118859

[67] Utsumi Y, Tanaka M, Kurotani A, Yoshida T, Mochida K, Matsui A, et al Cassava (Manihot esculenta. Journal of plant research. 2016;129(4):711–26. 10.1007/s10265-016-0828-x 27138000

[68] Danielson P. The cytochrome P450 superfamily: biochemistry, evolution and drug metabolism in humans. Current Drug Metabolism. 2002;3(6):561–97. 12369887 10.2174/1389200023337054

[69] Johnson SM, Lim F-L, Finkler A, Fromm H, Slabas AR, Knight MR. Transcriptomic analysis of Sorghum bicolor responding to combined heat and drought stress. BMC genomics. 2014;15(1):456. 24916767 10.1186/1471-2164-15-456 PMC4070570

[70] Zhang X, Liu T, Wei X, Qiu Y, Song J, Wang H, et al Expression patterns, molecular markers and genetic diversity of insect-susceptible and resistant Barbarea genotypes by comparative transcriptome analysis. BMC genomics. 2015;16:1–16. 10.1186/1471-2164-16-1 26126637 PMC4487577

[71] Dubey NK, Goel R, Ranjan A, Idris A, Singh SK, Bag SK, et al Comparative transcriptome analysis of Gossypium hirsutum L. in response to sap sucking insects: aphid and whitefly. BMC genomics. 2013;14(1):241–61. 23577705 10.1186/1471-2164-14-241 PMC3637549

[72] Kessler A, Baldwin IT. Plant responses to insect herbivory: the emerging molecular analysis. Annual Review of Plant Biology. 2002;53(1):299–328. 10.1146/annurev.arplant.53.100301.135207 12221978

[73] Broekgaarden C, Poelman EH, Steenhuis G, Voorrips RE, Dicke M, Vosman B. Responses of Brassica oleracea cultivars to infestation by the aphid Brevicoryne brassicae: an ecological and molecular approach. Plant, Cell & Environment. 2008;31(11):1592–605. 10.1111/j.1365-3040.2008.01871.x 18721268

[74] Beneventi MA, da Silva OB, de Sá MEL, Firmino AAP, de Amorim RMS, Albuquerque ÉVS, et al Transcription profile of soybean-root-knot nematode interaction reveals a key role of phythormones in the resistance reaction. BMC genomics. 2013;14(1):322–38. 23663436 10.1186/1471-2164-14-322 PMC3701510

[75] Gao L, Wang Y, Li Z, Zhang H, Ye J, Li G. Gene Expression Changes during the Gummosis Development of Peach Shoots in Response to Lasiodiplodia theobromae Infection Using RNA-Seq. Frontiers in physiology. 2016;7:1–12. 10.3389/fphys.2016.00001 27242544 PMC4861008

[76] Kumar S, Kanakachari M, Gurusamy D, Kumar K, Narayanasamy P, Kethireddy Venkata P, et al Genome‐wide transcriptomic and proteomic analyses of bollworm‐infested developing cotton bolls revealed the genes and pathways involved in the insect pest defence mechanism. Plant Biotechnology Journal. 2015;14: 1438–55. 10.1111/pbi.12508 PMC5066800 26799171

[77] Li H, Mahmood T, Antony G, Lu N, Pumphreys M, Gill B, et al The non-host pathogen Puccinia triticina elicits an active transcriptional response in rice. European Journal of Plant Pathology. 2016:1–17.

[78] Lo SF, Ho THD, Liu YL, Jiang MJ, Hsieh KT, Chen KT, et al Ectopic expression of specific GA2 oxidase mutants promotes yield and stress tolerance in rice. Plant Biotechnology Journal. 2016. 10.1111/pbi.12681 PMC5466439 27998028

[79] Wang Y, Zhou L, Yu X. Transcriptome profiling of Huanglongbing (HLB) tolerant and susceptible citrus plants reveals the role of basal resistance in HLB tolerance. Frontiers in Plant Science. 2016;7:1–13. 10.3389/fpls.2016.00001 27446161 PMC4923198

[80] Huttly AK, Phillips AL. Gibberellin-regulated expression in oat aleurone cells of two kinases that show homology to MAP kinase and a ribosomal protein kinase. Plant Molecular Biology. 1995;27(5):1043–52. 7766874 10.1007/BF00037031

[81] Synek L, Schlager N, Eliáš M, Quentin M, Hauser MT, Žárský V. AtEXO70A1, a member of a family of putative exocyst subunits specifically expanded in land plants, is important for polar growth and plant development. The Plant Journal. 2006;48(1):54–72. 10.1111/j.1365-313X.2006.02854.x 16942608 PMC2865999

[82] Garcia-Ranea J, Mirey G, Camonis J, Valencia A. p23 and HSP20/α‐crystallin proteins define a conserved sequence domain present in other eukaryotic protein families. FEBS Letters. 2002;529(2–3):162–7. 12372593 10.1016/s0014-5793(02)03321-5

[83] Lenfant N, Hotelier T, Bourne Y, Marchot P, Chatonnet A. Proteins with an alpha/beta hydrolase fold: relationships between subfamilies in an ever-growing superfamily. Chemico-Biological Interactions. 2013;203(1):266–8. 10.1016/j.cbi.2012.09.003 23010363

[84] Rawat N, Naga NC, Meenakshi SR, Nair S, Bentur JS. A novel mechanism of gall midge resistance in the rice variety Kavya revealed by microarray analysis. Functional & Integrative Genomics. 2012;12(2):249–64. 22447493 10.1007/s10142-012-0275-2

[85] Su P-H, Li H-m. Arabidopsis stromal 70-kD heat shock proteins are essential for plant development and important for thermotolerance of germinating seeds. Plant Physiology. 2008;146(3):1231–41. 10.1104/pp.107.114496 18192441 PMC2259073

[86] Hiremath PJ, Farmer A, Cannon SB, Woodward J, Kudapa H, Tuteja R, et al Large‐scale transcriptome analysis in chickpea (Cicer arietinum L.), an orphan legume crop of the semi‐arid tropics of Asia and Africa. Plant Biotechnology Journal. 2011;9(8):922–31. 10.1111/j.1467-7652.2011.00625.x PMC3437486 21615673

[87] Kawai Y, Ono E, Mizutani M. Evolution and diversity of the 2–oxoglutarate‐dependent dioxygenase superfamily in plants. The Plant Journal. 2014;78(2):328–43. 10.1111/tpj.12479 24547750

[88] Ambawat S, Sharma P, Yadav NR, Yadav RC. MYB transcription factor genes as regulators for plant responses: an overview. Physiology and Molecular Biology of Plants. 2013;19(3):307–21. 10.1007/s12298-013-0179-1 24431500 PMC3715649

[89] Roy S. Function of MYB domain transcription factors in abiotic stress and epigenetic control of stress response in plant genome. Plant signaling & behavior. 2016;11(1):1–7. 10.1080/15592324.2015.1117723 PMC4871670 26636625

[90] Klingler J, Creasy R, Gao L, Nair RM, Calix AS, Jacob HS, et al Aphid resistance in Medicago truncatula involves antixenosis and phloem-specific, inducible antibiosis, and maps to a single locus flanked by NBS-LRR resistance gene analogs. Plant Physiology. 2005;137(4):1445–55. 10.1104/pp.104.051243 15778464 PMC1088333

[91] Reymond P, Weber H, Damond M, Farmer EE. Differential gene expression in response to mechanical wounding and insect feeding in Arabidopsis. The Plant Cell. 2000;12(5):707–19. 10810145 10.1105/tpc.12.5.707 PMC139922

[92] Tao Y, Xie Z, Chen W, Glazebrook J, Chang H-S, Han B, et al Quantitative nature of Arabidopsis responses during compatible and incompatible interactions with the bacterial pathogen Pseudomonas syringae. The Plant Cell. 2003;15(2):317–30. 10.1105/tpc.007591 12566575 PMC141204

[93] Venu R, Madhav MS, Sreerekha M, Nobuta K, Zhang Y, Carswell P, et al Deep and comparative transcriptome analysis of rice plants infested by the beet armyworm (Spodoptera exigua) and water weevil (Lissorhoptrus oryzophilus). Rice. 2010;3(1):22–35.

[94] Zagrobelny M, Scheibye-Alsing K, Jensen NB, Møller BL, Gorodkin J, Bak S. 454 pyrosequencing based transcriptome analysis of Zygaena filipendulae with focus on genes involved in biosynthesis of cyanogenic glucosides. BMC genomics. 2009;10(1):1–15. 19954531 10.1186/1471-2164-10-574 PMC2791780

[95] Kang J, Park J, Choi H, Burla B, Kretzschmar T, Lee Y, et al Plant ABC transporters. American Society for Plant Biologist. 2011;153:1–25.

[96] Pitzschke A, Schikora A, Hirt H. MAPK cascade signalling networks in plant defence. Current Opinion in Plant Biology. 2009;12(4):421–6. 10.1016/j.pbi.2009.06.008 19608449

[97] Ballén-Taborda C, Plata G, Ayling S, Rodríguez-Zapata F, Becerra Lopez-Lavalle LA, Duitama J, et al Identification of cassava MicroRNAs under abiotic stress. International Journal of Genomics. 2013;2013:1–10. 10.1155/2013/857986 PMC3845235 24328029

[98] Bohorquez A, Becerra López-Lavalle LA, Bellotti AC, Tohme JM. Unraveling whitefly resistance in cassava (Manihot esculenta). 2012 Online, CIAT, Cali, Colombia.

[99] Fregene M, Matsumura H, Akano A, Dixon A, Terauchi R. Serial analysis of gene expression (SAGE) of host–plant resistance to the cassava mosaic disease (CMD). Plant Molecular Biology. 2004;56(4):563–71. 10.1007/s11103-004-3477-8 15630620

[100] Lopez C, Soto M, Restrepo S, Piégu B, Cooke R, Delseny M, et al Gene expression profile in response to Xanthomonas axonopodis pv. manihotis infection in cassava using a cDNA microarray. Plant Molecular Biology. 2005;57(3):393–410. 10.1007/s11103-004-7819-3 15830129

[101] Owiti J, Grossmann J, Gehrig P, Dessimoz C, Laloi C, Hansen MB, et al iTRAQ‐based analysis of changes in the cassava root proteome reveals pathways associated with post‐harvest physiological deterioration. The Plant Journal. 2011;67(1):145–56. 10.1111/j.1365-313X.2011.04582.x 21435052

[102] Utsumi Y, Tanaka M, Morosawa T, Kurotani A, Yoshida T, Mochida K, et al Transcriptome analysis using a high-density oligomicroarray under drought stress in various genotypes of cassava: an important tropical crop. DNA research. 2012;19(4):335–45. 10.1093/dnares/dss016 22619309 PMC3415295

[103] Wang W, Feng B, Xiao J, Xia Z, Zhou X, Li P, et al Cassava genome from a wild ancestor to cultivated varieties. Nature Communications. 2014;5:1–9. 10.1038/ncomms6110 PMC4214410 25300236

[104] Duffey SS, Stout MJ. Antinutritive and toxic components of plant defense against insects. Archives of Insect Biochemistry and Physiology. 1996;32(1):3–37.

[105] Steppuhn A, Baldwin IT. Resistance management in a native plant: nicotine prevents herbivores from compensating for plant protease inhibitors. Ecology Letters. 2007;10(6):499–511. 10.1111/j.1461-0248.2007.01045.x 17498149

