# Supplementary material for: The early transcriptome response of cassava (Manihot esculenta Crantz) to mealybug (Phenacoccus manihoti) feeding
Source: PLoS One. 2018 Aug 22;13(8):e0202541. doi: 10.1371/journal.pone.0202541 (PMC6105004; doi:10.1371/journal.pone.0202541)
Supplement: S1 Fig — Three independent biological replicates were performed. (PDF) [file pone.0202541.s001.pdf]

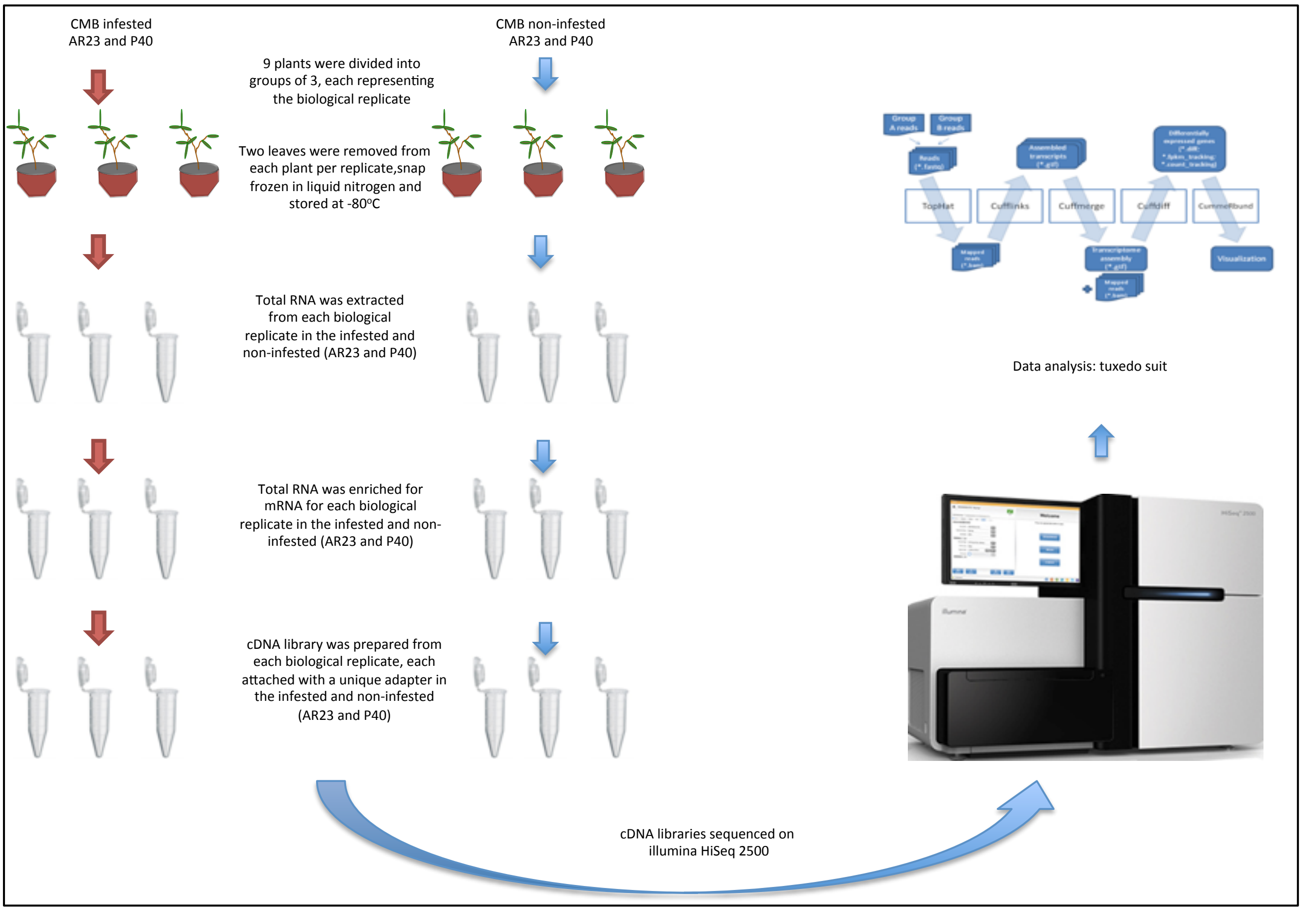

S1_Fig: The schematic representation of the experimental approach for RNA isolation and cDNA preparation for biological replicates of cassava genotypes infested with mealybugs. This figure illustrates one genotype at one time-point. This method was repeated for all the genotypes at deferent time-points (24 and 72 hours) post infestation with mealybugs compared to non-infested.
