# Supplementary material for: The early transcriptome response of cassava (Manihot esculenta Crantz) to mealybug (Phenacoccus manihoti) feeding
Source: PLoS One. 2018 Aug 22;13(8):e0202541. doi: 10.1371/journal.pone.0202541 (PMC6105004; doi:10.1371/journal.pone.0202541)
Supplement: S3 Fig — (PDF) [file pone.0202541.s003.pdf]

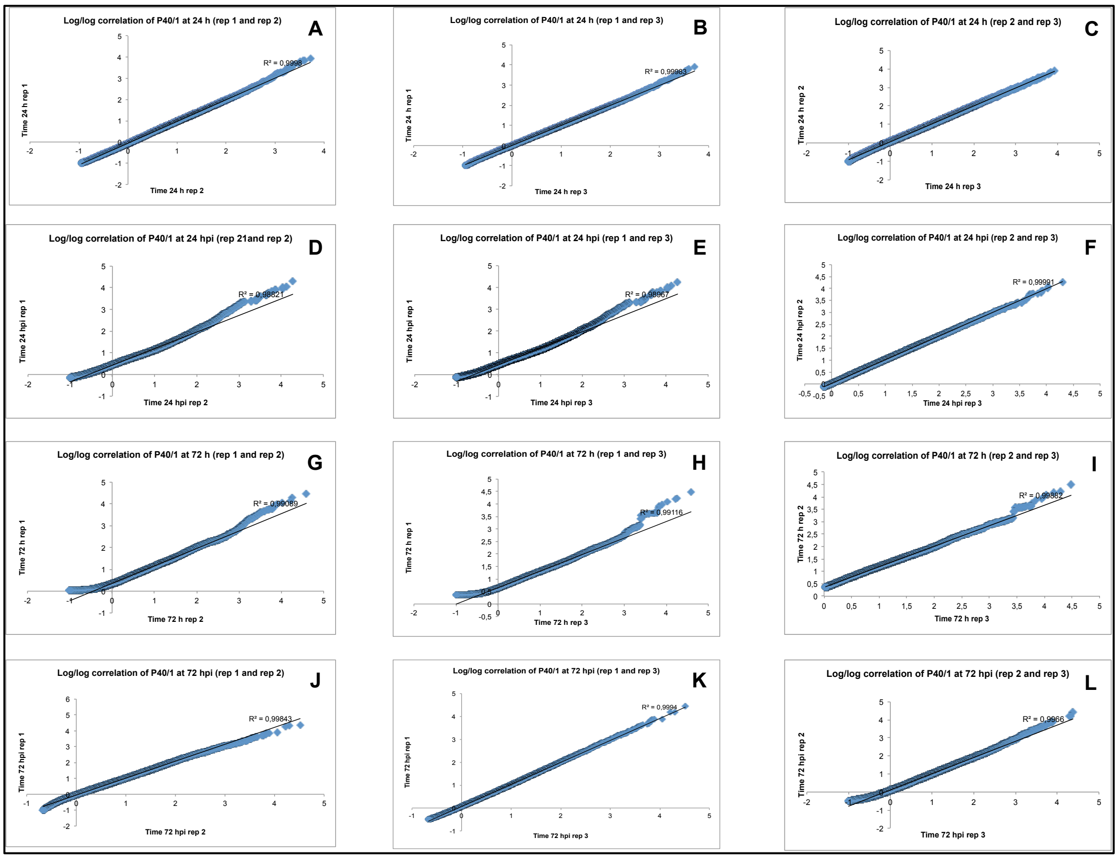

S3_Fig: The log/log correlations of P40/1 leaf samples infested with mealybugs (24 and 72 hours post infestation) compared to non-infested controls. Images A-C show correlations of 3 biological replicates at 24 h (non-infested control), D-F show correlations of 3 biological replicates at 24 hpi (infested at 24 h), G-I show correlations of 3 biological replicates at 72 h (non-infetsed control), while J-L show correlations of 3 biological replicates at 72 hpi (infested at 72 h).
