# Supplementary material for: The early transcriptome response of cassava (Manihot esculenta Crantz) to mealybug (Phenacoccus manihoti) feeding
Source: PLoS One. 2018 Aug 22;13(8):e0202541. doi: 10.1371/journal.pone.0202541 (PMC6105004; doi:10.1371/journal.pone.0202541)
Supplement: S4 Fig — (PDF) [file pone.0202541.s004.pdf]

CP data of housekeeping Genes:

	UBQ10	GTPb	EF1	ACTIN						
	HKG 1	HKG 2	HKG 3	HKG 4	HKG 5	HKG 6	HKG 7	HKG 8	HKG 9	HKG 10
n	12	12	12	12	0	0	0	0	0	0
geo Mean [CP]	31,77	24,73	30,14	35,89						
ar Mean [CP]	31,77	24,73	30,34	35,90						
min [CP]	30,60	24,25	25,12	34,82						
max [CP]	32,18	25,41	35,87	36,98						
std dev [± CP]	0,40	0,29	2,68	0,42						
CV [% CP]	1,25	1,16	8,83	1,16						
min [x-fold]	-2,25	-1,39	-32,50	-2,11						
max [x-fold]	1,33	1,60	52,99	2,12						
std dev [± x-fold]	1,32	1,22	6,40	1,33						

Pearson correlation coefficient (r)

vs.	HKG 1	HKG 2	HKG 3	HKG 4	HKG 5	HKG 6	HKG 7	HKG 8	HKG 9	HKG 10
HKG 2	0,570	-	-	-	-	-	-	-	-	-
p-value	0,053	-	-	-	-	-	-	-	-	-
HKG 3	0,681	0,790	-	-	-	-	-	-	-	-
p-value	0,015	0,002	-	-	-	-	-	-	-	-
HKG 4	-0,460	0,260	-0,053	-	-	-	-	-	-	-
p-value	0,132	0,415	0,868	-	-	-	-	-	-	-
HKG 5					-	-	-	-	-	-
p-value					-	-	-	-	-	-
HKG 6						-	-	-	-	-
p-value						-	-	-	-	-
HKG 7							-	-	-	-
p-value							-	-	-	-
HKG 8								-	-	-
p-value								-	-	-
HKG 9									-	-
p-value									-	-
HKG 10										-
p-value										-
BestKeeper vs.	HKG 1	HKG 2	HKG 3	HKG 4	HKG 5	HKG 6	HKG 7	HKG 8	HKG 9	HKG 10
coeff. of corr. [r]	0,708	0,858	0,990	0,021						
p-value	0,010	0,001	0,001	0,946						

Regression Analysis: HKG vs. BestKeeper

	UBQ10	GTPb	EF1	ACTIN						
	HKG 1 vs. BK	HKG 2 vs. BK	HKG 3 vs. BK	HKG 4 vs. BK	HKG 5 vs. BK	HKG 6 vs. BK	HKG 7 vs. BK	HKG 8 vs. BK	HKG 9 vs. BK	HKG 10 vs. BK
coeff. of corr. [r]	0,71	0,86	0,99	0,02						
coeff. of det. [r^2]	0,50	0,74	0,98	0,00						
intercept [CP]	21,68	16,10	-70,23	35,55						
slope [CP]	0,33	0,28	3,31	0,01						
SE [CP]	±0,375	±0,193	±0,536	±0,596						
p-value	0,010	0,001	0,001	0,946						
Power of HKG [x-fold]	1,26	1,22	9,92	1,01						

S4 Fig. Descriptive statistics of four candidate housekeeping genes (HKG) based on their crossing point (CP) values. BestKeeper index is computed together with the same descriptive parameters for four genes (UBQ10, GTPb, EF1 and Actin). Abbreviations: n: number of samples; geo Mean [CP]: the geometric mean of CP; ar Mean [CP]: the arithmetic mean of CP; Min [CP] and Max [CP]: the extreme values of CP; std dev [±CP]: the standard deviation of the CP; CV [%CP]: the coefficient of variance expressed as a percentage on the CP level; Min [x-fold] and Max [x-fold]: the extreme values of expression levels expressed as an absolute x-fold over- or under-regulation coefficient; std dev [± x-fold]: standard deviation of the absolute regulation coefficients.
