# Supplementary material for: The early transcriptome response of cassava (Manihot esculenta Crantz) to mealybug (Phenacoccus manihoti) feeding
Source: PLoS One. 2018 Aug 22;13(8):e0202541. doi: 10.1371/journal.pone.0202541 (PMC6105004; doi:10.1371/journal.pone.0202541)
Supplement: S5 Fig — (PDF) [file pone.0202541.s005.pdf]

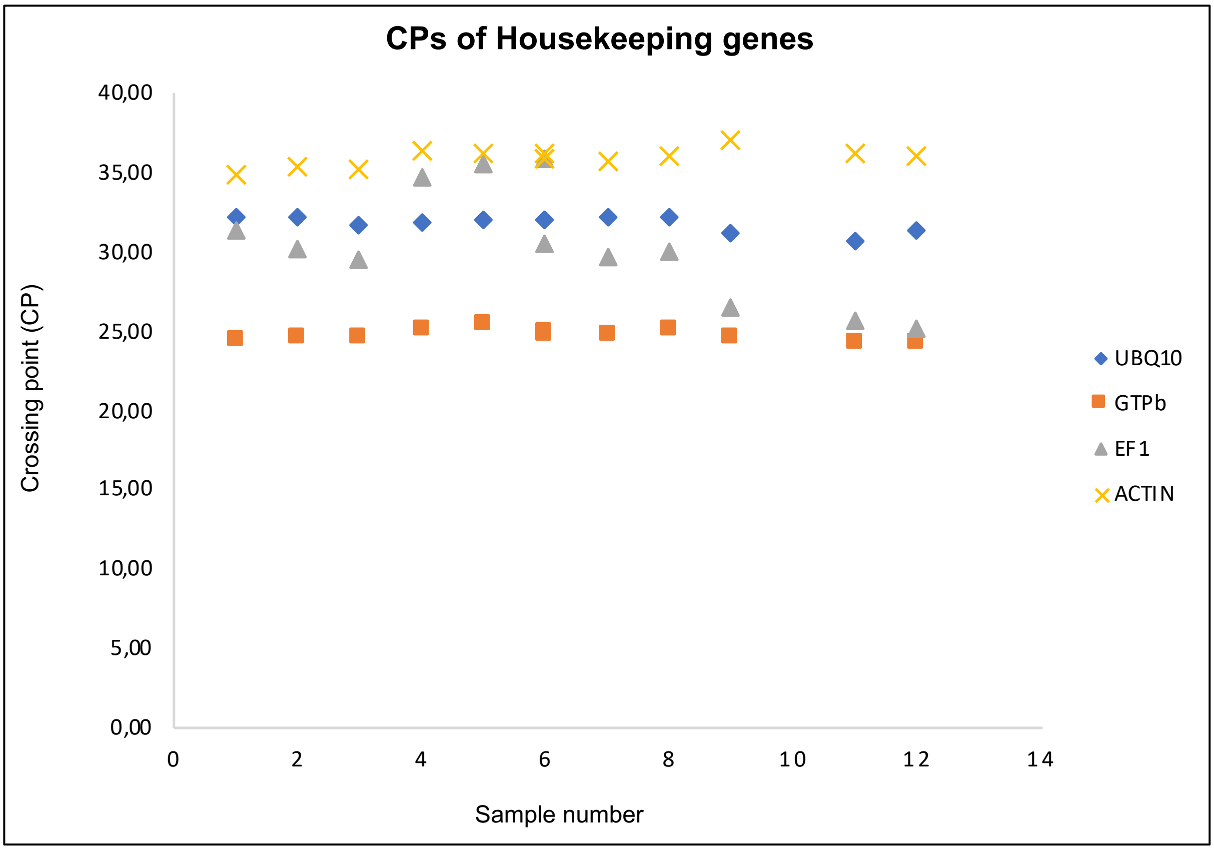

S5 Fig. Expression stability of the four candidate housekeeping genes (UBQ10, GTPb, EF1 and Actin) in cassava leaves. The figure illustrates the crossing points of each sample replicate for each gene.
