# Supplementary material for: The early transcriptome response of cassava (Manihot esculenta Crantz) to mealybug (Phenacoccus manihoti) feeding
Source: PLoS One. 2018 Aug 22;13(8):e0202541. doi: 10.1371/journal.pone.0202541 (PMC6105004; doi:10.1371/journal.pone.0202541)
Supplement: S1 Table — (PDF) [file pone.0202541.s006.pdf]

Table S1: Raw and trimmed reads generated per time point for each biological replicates and subsequent mapping to the cassava reference genome (*Mesculenta*_305_v6.1)

Genotype	Time (hours post infestation)	Infestation status	No, of raw reads	Trimmed reads	% Trimmed reads	Mapped reads	Mapped reads/million sequence reads
AR23,1 Biological replicate 3	24 h	Non-infested	21,444,473	19,110,834	89.1	16,921,128	885 420
	72 h	Non-infested	29,388,325	25,197,198	85.7	22,067,654	875 797
	24 hpi	Infested	42,212,956	41,500,026	98.3	30,032,612	723 538
	72 hpi	Infested	45,953,442	41,507,964	90.3	36,210,712	872 379
AR23,1 Biological replicate 2	24 h	Non-infested	29,065,379	25,218,186	86.7	23,190,120	919 579
	72 h	Non-infested	25,901,916	22,107,187	85.3	19,917,821	900 965
	24 hpi	Infested	40,000,000	36,490,056	91.2	32,054,329	878 440
	72 hpi	Infested	39,115,704	37,114,004	94.9	33,790,451	910 450
AR23,1 Biological replicate 3	24 h	Non-infested	37,694,984	33,107,823	87.7	29,108,912	879 215
	72 h	Non-infested	29,614,889	25,108,197	85.0	21,109,432	840 738
	24 hpi	Infested	38,453,272	34,989,258	90.9	32,157,979	919 081
	72 hpi	Infested	31,073,358	28,957,014	93.1	23,822,215	822 675
P40/1 Biological replicate 1	24 h	Non-infested	32,536,388	28,927,448	89.0	24,978,358	863 482
	72 h	Non-infested	29,514,034	25,216,917	85.4	22,421,686	889 152
	24 hpi	Infested	29,378,808	27,764,750	93.3	22,816,550	821 781
	72 hpi	Infested	23,424,700	20,774,916	88.7	19,526,920	843 657
P40/1 Biological replicate 2	24 h	Non-infested	26,937,496	23,928,218	89.0	20,684,329	864 432
	72 h	Non-infested	26,726,775	24,210,721	90.5	20,463,976	845 244
	24 hpi	Infested	35,954,764	34, 860,410	96.9	31,986,993	917 573
	72 hpi	Infested	33,394,144	32,224,992	95.0	25,158,790	780 722
P40/1 Biological replicate 3	24 h	Non-infested	22,016,380	20,219,012	91.0	17,989,129	889 713
	72 h	Non-infested	29,663,515	26,018,219	88.0	22,129,719	850 547
	24 hpi	Infested	33,346,560	28,740,636	86.1	24,109,731	838 872
	72 hpi	Infested	38,371,700	37,807,994	98.5	30,223,924	799 405
