# Supplementary material for: The early transcriptome response of cassava (Manihot esculenta Crantz) to mealybug (Phenacoccus manihoti) feeding
Source: PLoS One. 2018 Aug 22;13(8):e0202541. doi: 10.1371/journal.pone.0202541 (PMC6105004; doi:10.1371/journal.pone.0202541)
Supplement: S2 Table — (PDF) [file pone.0202541.s007.pdf]

S2 Table. List of AR23.1 differentially expressed genes in cassava leaves in response to mealybug infestation at 24 and 72 hours post infestation normalized against mock (non-infested).

Gene ID	GO ID	Gene annotation	Log2(fold_change) 24 hpi	P-value
Manes.01G059800	GO:0055114,GO:0020037,GO:0016705,GO:0005506	Cytochrome P450, family 86, subfamily A, polypeptide 8	6.07	0.00005
Manes.02G151200	-	Phosphate-responsive 1 family protein	6.01	0.00005
Manes.01G043500	GO:0055114,GO:0016491	2-oxoglutarate (2OG) and Fe(II)-dependent oxygenase superfamily protein	5.45	0.00005
Manes.17G058800	-	Ucharacterized protein	5.33	0.00005
Manes.17G070900	GO:0003824	Camelliol C synthase 1	5.25	0.00005
Manes.02G189200	GO:0016788	GDSL-like Lipase/Acylhydrolase superfamily protein	4.72	0.00005
Manes.09G175800	-	Hypothetical protein	4.54	0.0002
Manes.01G200000	GO:0009611,GO:0004867,GO:0055114,GO:0020037,GO:0016705,GO:0005506	Cytochrome P450, family 94, subfamily B, polypeptide 3	4.39	0.00005
Manes.04G050300	-	RHO GTPASE Activating protein	4.2.0	0.00005
Manes.01G020600	GO:0030983,GO:0006298,GO:0005524	MUTS homolog 2	4.18	0.00015
Manes.02G086300	GO:0016829,GO:0010333,GO:0008152,GO:0000287	Terpene synthase 21	4.17	0.00005
Manes.01G243800	GO:0015743	Aluminium activated malate transporter family protein	4.07	0.0001
Manes.15G094100	GO:0048544	D-mannose binding lectin protein with Apple-like carbohydrate-binding domain	3.93	0.00015
Manes.08G103300,Manes.08G103400	-	Hypothetical protein	3.82	0.0001
Manes.01G147500	-	Myb domain protein 4	3.79	0.00015
Manes.07G061600	GO:0009958	Phototropic-responsive NPH3 family protein	3.69	0.00015
Manes.02G124600	-	HSP20-like chaperones superfamily protein	3.69	0.00005
Manes.01G158000	GO:0055085,GO:0016021	Auxin efflux carrier family protein	3.54	0.0001
Manes.01G067000	GO:0055085,GO:0016020,GO:0015297,GO:0015238,GO:0006855	MATE efflux family protein	3.37	0.00005
Manes.17G078600	-	CLAVATA3/ESR-RELATED 16	3.35	0.0001
Manes.01G076500	GO:0004575,GO:0004564	Glycosyl hydrolases family 32 protein	3.21	0.00005
Manes.01G231100	GO:0055114,GO:0016491	Gibberellin 2-oxidase 6	3.06	0.00005
Manes.02G051100	GO:0055085,GO:0016020,GO:0006811,GO:0005216	Cyclic nucleotide-regulated ion channel family protein	2.88	0.00005
Manes.06G136100	GO:0004857	Plant invertase/pectin methylesterase inhibitor superfamily protein	2.59	0.00005
Manes.06G131100	GO:0006355,GO:0003700	Ethylene responsive element binding factor 1	2.41	0.0002
Manes.09G040500	-	Alpha/beta-Hydrolases superfamily protein	2.37	0.0001
Manes.01G274500	GO:0006629	Alpha/beta-Hydrolases superfamily protein	2.17	0.00005
Manes.11G057100	GO:0005515	Leucine-rich repeat (LRR) family protein	2.10	0.00005
Manes.14G096200	GO:0005515	RNI-like superfamily protein	2.00	0.00005
Manes.05G143800	-	Expansin 11	1.89	0.00005
Manes.03G048300	GO:0005515,GO:0006468,GO:0004672	Leucine-rich repeat transmembrane protein kinase family protein	1.68	0.00005
Manes.15G094300	GO:0048544	D-mannose binding lectin protein with Apple-like carbohydrate-binding domain	1.58	0.00005
Manes.01G142200	GO:0005515	Leucine-rich repeat receptor-like protein kinase family protein	1.58	0.00005
Manes.01G273700	GO:0043565,GO:0006355,GO:0003700	WRKY family transcription factor	1.58	0.00005
Manes.05G132500	GO:0008017,GO:0007018,GO:0005524,GO:0003777,GO:0005871	ATP binding microtubule motor family protein	1.37	0.00005

Manes.01G235800	-	Myb domain protein 55	1.37	0.00005
Manes.07G059300	GO:0005975,GO:0004553	Glycosyl hydrolase superfamily protein	1.35	0.0001
Manes.10G105100	GO:0016887,GO:0005524,GO:0016020	White-brown complex homolog protein 11	1.16	0.00015
Manes.03G079300	GO:0008270,GO:0005515	RING/U-box superfamily protein	0.71	0.00005
Manes.13G137200	GO:0045735	RmlC-like cupins superfamily protein	0.24	0.00005
Manes.02G026300	GO:0055114,GO:0020037,GO:0006979,GO:0004601	Peroxidase superfamily protein	-0.12	0.00005
Manes.16G045900	GO:0006468,GO:0005524,GO:GO:0004672	MAP kinase kinase 5	-1.91	0.00005
Manes.11G052600	-	Late embryogenesis abundant protein (LEA) family protein	-2.61	0.0001
Manes.01G129800	-	Alpha-crystallin domain 32.1	-2.74	0.0001
Manes.S110500	-	Hypothetical protein	-3.11	0.00005
Manes.14G051800	GO:0055114,GO:0020037,GO:0016705,GO:0005506	Cytochrome P450, family 94, subfamily B, polypeptide 3	-3.25	0.00005
Manes.02G081800	GO:0005515	Glutathione S-transferase F11	-3.34	0.00005
Manes.02G023600	-	Protein of unknown function (DUF567)	-3.46	0.00005
Manes.02G025100	-	Osmotin 34	-3.53	0.00005
Manes.14G143500	-	Heat shock protein 70B	-3.53	0.0001
Manes.01G252200	GO:0043565,GO:0006355,GO:0003700	WRKY DNA-binding protein 56	-3.57	0.00005
Manes.01G114100	-	Gamma interferon responsive lysosomal thiol (GILT) reductase family protein	-3.76	0.00005
Manes.02G005800	GO:0016810,GO:0006807	Carbon-nitrogen hydrolase family protein	-4.03	0.00005
Manes.01G187700	GO:0046983	Basic helix-loop-helix (bHLH) DNA-binding family protein	-4.10	0.00015
Manes.02G008500	-	Galactose-binding protein	-4.15	0.00005
Manes.02G015400	GO:0009378,GO:0006310,GO:0006281	P-loop containing nucleoside triphosphate hydrolases superfamily protein	-4.17	0.00005
Manes.01G110800	GO:0008270,GO:0005975,GO:0004476,GO:0009298	Mannose-6-phosphate isomerase, type I	-4.17	0.00005
Manes.01G178000	GO:0055114,GO:0020037,GO:0016705,GO:0005506	Cytochrome P450 superfamily protein	-4.19	0.00015
Manes.02G040000	GO:0016021,GO:0016020,GO:0022857	Nodulin MtN21 /EamA-like transporter family protein	-4.19	0.00005
Manes.01G268700	-	Bifunctional inhibitor/lipid-transfer protein/seed storage 2S albumin superfamily protein	-4.46	0.00005
Manes.11G034200	GO:0016887,GO:0005524,GO:0016020	ABC-2 and Plant PDR ABC-type transporter family protein	-4.47	0.00005
Manes.03G043600	GO:0004857	Plant invertase/pectin methylesterase inhibitor superfamily protein	-4.53	0.0001
Manes.01G020700	-	TCP family transcription factor	-4.80	0.00005
Manes.01G250800	GO:0051087	BCL-2-associated athanogene 6	-4.82	0.0003
Manes.02G174100	-	Nodulin MtN3 family protein	-4.82	0.00005
Manes.03G061200	-	Tetraspanin family protein	-4.90	0.00005
Manes.01G058100	-	Pectin lyase-like superfamily protein	-4.95	0.00005
Manes.01G082400	GO:0030983,GO:0006298,GO:0005524	MUTS-like protein 4	-5.11	0.00005
Manes.01G013000	GO:0006468,GO:0005524,GO:0004672	Wall-associated kinase family protein	-5.34	0.00005
Manes.01G138100	GO:0005975,GO:0004553	Glycosyl hydrolase superfamily protein	-5.53	0.00005
Manes.01G161800	GO:0055085,GO:0016021	Cation exchanger 3	-5.63	0.00005
Manes.01G235200	-	Pectin lyase-like superfamily protein	-5.63	0.00005
Manes.03G018600	GO:0016887,GO:0005524,GO:0016020	Pleiotropic drug resistance 12	-5.65	0.00015
Manes.03G062400	GO:0005515	Transducin/WD40 repeat-like superfamily protein	-5.65	0.00025
Manes.15G189500	-	HSP20-like chaperones superfamily protein	-5.73	0.00015
Manes.02G084300	-	Phy rapidly regulated 1	-5.74	0.00005
Manes.01G087800	-	Expansin-like B1	-5.74	0.00005
Manes.02G109900	-	Protein of unknown function	-5.86	0.00015

		(DUF630 and DUF632)		
Manes.01G031800	GO:0008270	DHHC-type zinc finger family protein	-5.87	0.0001
Manes.02G001500	GO:0055114,GO:0016491,GO:0008270	GroES-like zinc-binding dehydrogenase family protein	-5.91	0.00005
Manes.01G070800	GO:0006355,GO:0005634,GO:0005524,GO:0032502,GO:0006351	Growth-regulating factor 2	-5.95	0.00005
Manes.01G254100	GO:0016757,GO:0016020	Protein of unknown function (DUF604)	-5.97	0.00025
Manes.02G106500	GO:0016021,GO:0006952	Seven transmembrane MLO family protein	-6.03	0.00005
Manes.03G120700	GO:0045454,GO:0005681,GO:0000398	mRNA splicing factor, thioredoxin-like U5 snRNP	-6.11	0.0001
Manes.01G070200	GO:0055114,GO:0016491	Leucoanthocyanidin dioxygenase	-6.11	0.00005
Manes.01G173300	GO:0006468,GO:0004672	Protein kinase superfamily protein	-6.13	0.00005
Manes.02G104600	-	GAST1 protein homolog 1	-6.34	0.0002
Manes.01G013100	-	PLAC8 family protein	-6.57	0.00005
Manes.03G066400	GO:0016021,GO:0015116,GO:0008272,GO:0055085,GO:0016020,GO:0008271,GO:1902358	Sulfate transporter 3;5	-6.58	0.0001
Manes.02G099700	GO:0016787	purple acid phosphatase 25	-6.64	0.00005
Manes.15G154400	-	HSP20-like chaperones superfamily protein	-6.80	0.00005
Manes.S053400	-	HSP20-like chaperones superfamily protein	-7.27	0.00005

**Gene ID**	**GO ID**	**Gene annotation**	**Log2(fold_change) 72 hpi**	**P-value**
Manes.01G200000	GO:0009611,GO:0004867,GO:0055114,GO:0020037,GO:0016705,GO:0005506	cytochrome P450, family 94, subfamily B, polypeptide 3	6.94	0.00005
Manes.03G040000	-	Mob1/phocein family protein	6.83	0.00005
Manes.02G115200	GO:0055085,GO:0016021	Auxin efflux carrier family protein	5.93	0.00005
Manes.01G043500	GO:0055114,GO:0016491	2-oxoglutarate (2OG) and Fe(II)-dependent oxygenase superfamily protein	5.88	0.00005
Manes.02G126100	-	Glutathione S-transferase tau 7	5.69	0.00005
Manes.01G264600	GO:0055114,GO:0020037,GO:0016705,GO:0005506	Cytochrome P450, family 704, subfamily A, polypeptide 2	5.68	0.00005
Manes.13G043400	-	Glycine-rich protein 5-like	5.51	0.00005
Manes.02G208500	GO:0006355,GO:0003700	Integrase-type DNA-binding superfamily protein	5.47	0.00005
Manes.01G070800	GO:0006355,GO:0005634,GO:0005524,GO:0032502,GO:0006351	Growth-regulating factor 2	5.08	0.00005
Manes.02G032300	GO:0055085,GO:0016020,GO:0015297,GO:0015238,GO:0006855	MATE efflux family protein	5.03	0.00005
Manes.06G136300	GO:0004857	Plant invertase/pectin methylesterase inhibitor superfamily protein	5.02	0.00005
Manes.01G189000	GO:0043565,GO:0006355,GO:0003700	WRKY DNA-binding protein 72	5.00	0.00005
Manes.03G056100	GO:0006355,GO:0003700	Integrase-type DNA-binding superfamily protein	4.97	0.00005
Manes.02G105300	-	Pathogenesis-related thaumatin superfamily protein	4.80	0.00005
Manes.02G002200	-	Alpha/beta-Hydrolases superfamily protein	4.56	0.00005
Manes.17G058800	-	Uncharacterized protein	4.55	0.0001
Manes.02G145900	GO:0006508,GO:0004190	Eukaryotic aspartyl protease family protein	4.54	0.00005
Manes.10G146700	GO:0055114,GO:0020037,GO:0016705,GO:0005506	Cytochrome P450, family 705, subfamily A, polypeptide 27	4.37	0.0001
Manes.02G086300	GO:0016829,GO:0010333,GO:0008152,GO:0000287	Terpene synthase 21	4.19	0.00005
Manes.01G231100	GO:0055114,GO:0016491	Gibberellin 2-oxidase 6	3.81	0.00005
Manes.01G076500	GO:0004575,GO:0004564	Glycosyl hydrolases family 32 protein	3.79	0.00005
Manes.02G086200	GO:0016829,GO:0010333,GO:0008152,GO:0000287	Terpenoid cyclases/Protein prenyltransferases superfamily protein	3.77	0.00005
Manes.02G150900	-	Phosphate-responsive 1 family protein	3.62	0.00005
Manes.08G103300,Manes.08G103400	-	Hypothetical protein	3.53	0.00005

Manes.01G129500	GO:0005515,GO:0006468,GO:0005524,GO:0004672	Leucine-rich repeat protein kinase family protein	3.32	0.00005
Manes.01G231200	GO:0042545,GO:0030599,GO:0005618	Pectin lyase-like superfamily protein	3.30	0.00005
Manes.S053400	-	HSP20-like chaperones superfamily protein	3.30	0.00005
Manes.02G189200	GO:0016788	GDSL-like Lipase/Acylhydrolase superfamily protein	3.01	0.00005
Manes.06G154000	GO:0016887,GO:0005524,GO:0016020	ABC-2 type transporter family protein	2.95	0.00005
Manes.02G124600	-	HSP20-like chaperones superfamily protein	2.83	0.00005
Manes.01G274500	GO:0006629	Alpha/beta-Hydrolases superfamily protein	2.79	0.00015
Manes.15G130200	GO:0009607,GO:0006952	Polyketide cyclase/dehydrase and lipid transport superfamily protein	2.71	0.0001
Manes.15G015600	GO:0045454,GO:0015035,GO:0009055	Glutaredoxin family protein	2.58	0.00005
Manes.01G147500	-	Myb domain protein 4	2.44	0.00005
Manes.11G080400	-	dsRNA-binding protein 5	2.31	0.0001
Manes.01G126300	GO:0004866	Kunitz trypsin inhibitor 1	2.15	0.00005
Manes.15G130300	GO:0009607,GO:0006952	MLP-like protein 43	1.87	0.0002
Manes.04G120100	GO:0055114,GO:0016491	2-oxoglutarate (2OG) and Fe(II)-dependent oxygenase superfamily protein	1.81	0.00005
Manes.01G188800	-	Hypothetical protein	1.39	0.00005
Manes.15G015500	GO:0045454,GO:0015035,GO:0009055	Thioredoxin superfamily protein	0.86	0.00005
Manes.03G012600	GO:0016798,GO:0016020	glucuronidase 3	0.41	0.00005
Manes.02G040700	-	Protein of unknown function, DUF547	0.39	0.00005
Manes.10G105100	GO:0016887,GO:0005524,GO:0016020	White-brown complex homolog protein 11	-0.30	0.00005
Manes.07G059300	GO:0005975,GO:0004553	Glycosyl hydrolase superfamily protein	-0.61	0.0001
Manes.10G106000	-	homeobox protein 31	-0.84	0.00025
Manes.01G041100	GO:0005515	Disease resistance family protein / LRR family protein	-0.85	0.00005
Manes.14G096200	GO:0005515	RNI-like superfamily protein	-1.22	0.00005
Manes.05G143800	-	expansin 11	-1.30	0.0001
Manes.03G138800	GO:0055114,GO:0020037,GO:0016705,GO:0005506	Cytochrome P450, family 71, subfamily B, polypeptide 34	-1.36	0.0001
Manes.02G124400	-	Protein of unknown function (DUF3527)	-1.54	0.0001
Manes.14G051800	GO:0055114,GO:0020037,GO:0016705,GO:0005506	Cytochrome P450, family 94, subfamily B, polypeptide 3	-1.60	0.00005
Manes.07G061600	GO:0009958	Phototropic-responsive NPH3 family protein	-3.01	0.00005
Manes.01G108600	GO:0016020	Uncharacterized protein family (UPF0016)	-3.23	0.00015
Manes.15G148400	-	Bifunctional inhibitor/lipid-transfer protein/seed storage 2S albumin superfamily protein	-3.27	0.0001
Manes.02G040000	GO:0016021,GO:0016020,GO:0022857	Nodulin MtN21 /EamA-like transporter family protein	-3.31	0.00015
Manes.02G076300	GO:0006412,GO:0005840,GO:0005622,GO:0003735	Nucleic acid-binding, OB-fold-like protein	-3.35	0.00005
Manes.17G113800	-	RmlC-like cupins superfamily protein	-3.46	0.00005
Manes.01G086900	GO:0055085,GO:0016021	Major facilitator superfamily protein	-3.51	0.00005
Manes.02G205700	GO:0007165,GO:0005515,GO:0043531	Disease resistance protein (TIR-NBS-LRR class), putative	-3.57	0.00005
Manes.02G039500	GO:0042545,GO:0030599,GO:0005618	Pectin lyase-like superfamily protein	-3.60	0.0001
Manes.01G040000	GO:0005975,GO:0004553	O-Glycosyl hydrolases family 17 protein	-3.82	0.0001
Manes.01G202400	-	Class I glutamine amidotransferase-like superfamily protein	-4.12	0.00005
Manes.02G017000	GO:0005975,GO:0004553	Glycosyl hydrolase 9B15	-4.30	0.00005
Manes.03G021100	GO:0055085,GO:0022857,GO:0016021	Polyol/monosaccharide transporter 5	-4.30	0.00005
Manes.01G142200	GO:0005515	Leucine-rich repeat receptor-like protein kinase family protein	-4.37	0.00005

Manes.02G170200	GO:0005509,GO:0006468,GO:0005524,GO:0004672	Wall associated kinase 3	-4.51	0.00005
Manes.02G054300	GO:0016758,GO:0008152	UDP-Glycosyltransferase superfamily protein	-4.52	0.00005
Manes.02G169700	GO:0016021	Nodulin MtN3 family protein	-4.57	0.00045
Manes.01G065500	GO:0055114,GO:0020037,GO:0006979,GO:0004601	Peroxidase superfamily protein	-4.68	0.0004
Manes.16G016100,Manes.16G016200,Manes.16G016300	GO:0016829,GO:0010333,GO:0008152,GO:0000287	Terpene synthase 14	-4.83	0.00005
Manes.03G027900	-	Oxidative stress 3	-4.85	0.0001
Manes.03G097700	GO:0006468,GO:0005524,GO:0004672	MAP kinase kinase 5	-4.86	0.00005
Manes.02G044700	-	Expansin-like B1	-4.9	0.00005
Manes.02G108300	GO:0008234,GO:0006508	Senescence-associated gene 12	-4.92	0.00005
Manes.03G061200	-	Tetraspanin family protein	-4.94	0.00025
Manes.02G025800	GO:0055114,GO:0020037,GO:0006979,GO:0004601	Peroxidase superfamily protein	-4.94	0.00005
Manes.01G002800	GO:0005975,GO:0004553	Cellulase 2	-4.96	0.0002
Manes.03G032500	GO:0055114,GO:0016491	ACC oxidase 1	-5	0.00005
Manes.01G203500	-	Calmodulin-like 11	-5.11	0.00005
Manes.01G047000	-	Alpha/beta-Hydrolases superfamily protein	-5.17	0.00005
Manes.02G071200	GO:0008152,GO:0003824	Cytochrome P450, family 77, subfamily A, polypeptide 5 pseudogene	-5.17	0.00005
Manes.18G024300	-	RAD-like 6	-5.41	0.00005
Manes.03G062400	GO:0005515	Transducin/WD40 repeat-like superfamily protein	-5.46	0.00005
Manes.02G067700	-	Regulator of Vps4 activity in the MVB pathway protein	-5.47	0.00005
Manes.01G067400	GO:0006355,GO:0003700	C-repeat-binding factor 4	-5.89	0.00005
Manes.01G002600	-	Alpha/beta-Hydrolases superfamily protein	-5.92	0.00005
Manes.01G078200	-	Bifunctional inhibitor/lipid-transfer protein/seed storage 2S albumin superfamily protein	-6.41	0.0001
Manes.02G151300	-	Phosphate-responsive 1 family protein	-6.70	0.00025
Manes.03G063600	GO:0006355,GO:0005634,GO:0003677	S1FA-like DNA-binding protein	-6.76	0.00025
Manes.02G089000	GO:0006355,GO:0003677	NAC domain containing protein 42	-6.79	0.0001
Manes.01G070200	GO:0055114,GO:0016491	Leucoanthocyanidin dioxygenase	-6.82	0.00025
Manes.02G025400	-	Uncharacterised protein family (UPF0497)	-6.92	0.00005
Manes.01G087800	-	Expansin-like B1	-6.95	0.00005
Manes.02G099700	GO:0016787	purple acid phosphatase 25	-6.96	0.0002
Manes.01G067500	GO:0006355,GO:0003700	C-repeat-binding factor 4	-6.96	0.00005
Manes.01G178000	GO:0055114,GO:0020037,GO:0016705,GO:0005506	Cytochrome P450 superfamily protein	-6.99	0.00005
Manes.02G148300	GO:0016021,GO:0005215	purine permease 3	-7.09	0.0001
Manes.02G025100	-	osmotin 34	-7.51	0.00005
Manes.02G015400	GO:0009378,GO:0006310,GO:0006281	P-loop containing nucleoside triphosphate hydrolases superfamily protein	-7.94	0.0001

**Gene ID**	**GO ID**	**Gene annotation**	**Log2(fold_change) 24 hpi vs 72 hpi**	
Manes.01G178000	GO:0055114,GO:0020037,GO:0016705,GO:0005506	Cytochrome P450 superfamily protein	6.81	0.00005
Manes.02G099700	GO:0016787	Purple acid phosphatase 25	6.69	0.00005
Manes.02G151000	-	Phosphate-responsive 1 family protein	6.54	0.00005
Manes.01G236500	-	Germin-like protein 10	5.79	0.00005
Manes.02G150900	-	Phosphate-responsive 1 family protein	5.75	0.00005
Manes.02G170600	GO:0006468,GO:0004672	Wall associated kinase 3	5.75	0.00005
Manes.01G121300	GO:0016567,GO:0004842	ARM repeat superfamily protein	5.70	0.00005
Manes.01G235200	-	Pectin lyase-like superfamily protein	5.60	0.0001
Manes.03G044100	GO:0006355,GO:0003700	Integrase-type DNA-binding superfamily protein	5.26	0.00005
Manes.01G047200	GO:0043565,GO:0006355,GO:0003700	WRKY family transcription factor	4.86	0.00005

Manes.02G075400	-	Calmodulin like 23	4.64	0.00005
Manes.02G150200	GO:0006468,GO:0005524,GO:0004672,GO:0005515	Leucine-rich repeat receptor-like protein kinase family protein	4.57	0.00005
Manes.02G130000	GO:0008270,GO:0005515	Zinc finger (C3HC4-type RING finger) family protein	4.51	0.00005
Manes.01G085400	GO:0006355,GO:0003700	Ethylene responsive element binding factor 1	4.14	0.00005
Manes.01G260700	GO:0005515	BON association protein 2	4.08	0.0001
Manes.S053400	GO:0006529,GO:0004066	HSP20-like chaperones superfamily protein	4.01	0.0001
Manes.02G146000	-	Dicarboxylate carrier 2	3.92	0.0001
Manes.12G152600	-	MAP kinase substrate 1	3.67	0.00005
Manes.14G006300	GO:0005515	CCT motif family protein	3.22	0.00005
Manes.16G132600	GO:0006508,GO:0004190	Eukaryotic aspartyl protease family protein	2.95	0.0001
Manes.15G121600	GO:0055114,GO:0016491	Gibberellin 2-oxidase	2.95	0.00005
Manes.02G110100	GO:0008270,GO:0005515	RING/U-box superfamily protein	2.87	0.00005
Manes.15G122400	-	Nine-cis-epoxycarotenoid dioxygenase 5	2.81	0.00005
Manes.15G035200	GO:0007165	CBL-interacting protein kinase 7	2.72	0.00005
Manes.13G112500	GO:0008270,GO:0006355,GO:0005634,GO:0004402,GO:0003712,GO:0005515	BTB and TAZ domain protein 4	2.48	0.00005
Manes.01G192100	GO:0045454,GO:0015035,GO:0006662	Thioredoxin H-type 7	2.47	0.00005
Manes.01G267100	-	Vegetative cell wall protein gp1-like	2.41	0.00005
Manes.02G039000	GO:0006508,GO:0004190	Eukaryotic aspartyl protease family protein	2.38	0.00005
Manes.05G061300	GO:0006950	Late embryogenesis abundant 3 (LEA3) family protein	2.28	0.00005
Manes.01G138500,Manes.01G138600	GO:0008168	S-adenosyl-L-methionine-dependent methyltransferases superfamily protein	2.18	0.00005
Manes.02G092000	GO:0008270,GO:0005634	DWNN domain, a CCHC-type zinc finger	1.77	0.00005
Manes.01G123500	GO:0046872	Regulator of chromosome condensation (RCC1) family with FYVE zinc finger domain	1.23	0.0001
Manes.01G241000	GO:0016747,GO:0008610,GO:0016020,GO:0006633	3-ketoacyl-CoA synthase 1	-1.61	0.0001
Manes.04G140500	-	Glycosyl hydrolases family 32 protein	0.23	0.00005
Manes.06G004300	GO:0016887,GO:0005524,GO:0016020	ABC-2 type transporter family protein	-0.41	0.00005
Manes.15G149600	-	Myb domain protein 24	-1.15	0.00005
Manes.15G060200	-	Putative uncharacterized protein DDB_G0290521	-1.36	0.0001
Manes.01G089900	-	Bifunctional inhibitor/lipid-transfer protein/seed storage 2S albumin superfamily protein	-1.38	0.00005
Manes.01G189000	GO:0043565,GO:0006355,GO:0003700	WRKY DNA-binding protein 72	-1.91	0.0001
Manes.15G015600	GO:0045454,GO:0015035,GO:0009055	Glutaredoxin family protein	-1.98	0.0001
Manes.04G121600	-	Galactose oxidase/kelch repeat superfamily protein	-2.10	0.0001
Manes.09G062300	GO:0016020,GO:0006810,GO:0005215	Gamma tonoplast intrinsic protein	-2.10	0.00005
Manes.06G007600	-	Calcium-binding EF-hand family protein	-2.23	0.00005
Manes.04G013300	GO:0008270,GO:0005515	CHY-type/CTCHY-type/RING-type Zinc finger protein	-2.29	0.00005
Manes.01G233000	GO:0006508	tolB protein-related	-2.29	0.0001
Manes.05G096400	-	PAS/LOV protein B	-2.33	0.00005
Manes.10G071700	GO:0055114,GO:0016491,GO:0051536,GO:0020037	Nitrite reductase 1	-2.33	0.00005
Manes.18G036200	GO:0005509	Calcium-binding EF-hand family protein	-2.35	0.0001
Manes.04G094700	GO:0071805,GO:0016020,GO:0015079	K+ uptake transporter 3	-2.37	0.00005
Manes.15G085500	-	Aluminium induced protein with YGL and LRDR motifs	-2.40	0.00005
Manes.08G067800	-	Bifunctional inhibitor/lipid-transfer	-2.47	0.0001

		protein/seed storage 2S albumin superfamily protein		
Manes.11G067500	GO:0003824,GO:0006470,GO:0004722	Heat shock protein 70B	-2.53	0.00005
Manes.02G128000	GO:0045454,GO:0015035,GO:0009055	Highly ABA-induced PP2C gene 3	-2.54	0.00005
Manes.15G015500	GO:0006529,GO:0004066	Thioredoxin superfamily protein	-2.61	0.0001
Manes.02G212800	GO:0008081,GO:0006629	Glutamine-dependent asparagine synthase 1	-2.61	0.00005
Manes.04G089800	-	Senescence-related gene 3	-2.62	0.0001
Manes.11G067600	-	Heat shock protein 70B	-2.63	0.00005
Manes.11G068500	-	Chaperone DnaJ-domain superfamily protein	-2.67	0.00005
Manes.01G119000	-	HSP20-like chaperones superfamily protein	-2.67	0.00005
Manes.09G112800	-	RNI-like superfamily protein	-2.71	0.00005
Manes.17G076700	GO:0016829,GO:0010333,GO:0008152,GO:0000287	Chaperone DnaJ-domain superfamily protein	-2.85	0.0001
Manes.04G052400	GO:0004866	Late embryogenesis abundant (LEA) hydroxyproline-rich glycoprotein family	-2.94	0.00005
Manes.S099100	-	Terpene synthase-like sequence-1,8-cineole	-2.99	0.00005
Manes.01G126300	-	Kunitz trypsin inhibitor 1	-3.04	0.00005
Manes.07G123200	GO:0020037,GO:0017004	HSP20-like chaperones superfamily protein	-3.11	0.00005
Manes.02G051000	-	Cyclic nucleotide-regulated ion channel family protein	-3.15	0.00005
Manes.07G086000	-	Cytochrome C assembly protein	-3.16	0.0001
Manes.10G105900	GO:0008080	HSP20-like chaperones superfamily protein	-3.28	0.00005
Manes.08G103000	-	GRAM domain family protein	-3.30	0.00005
Manes.03G040200	GO:0004869	Acyl-CoA N-acyltransferases (NAT) superfamily protein	-3.30	0.0002
Manes.15G028300	-	heat-shock protein 70T-2	-3.32	0.00015
Manes.09G039400	GO:0043565,GO:0006355,GO:0003700	Cystatin/monellin superfamily protein	-3.42	0.00005
Manes.16G048700	GO:0055114,GO:0020037,GO:0006979,GO:0004601	HSP20-like chaperones superfamily protein	-3.46	0.0001
Manes.02G061900	GO:0070569,GO:0008152	Basic leucine-zipper 42	-3.48	0.00005
Manes.08G002700	-	Ascorbate peroxidase 2	-3.52	0.0001
Manes.02G169300,Manes.02G169400	GO:0006468,GO:0005524,GO:0004672	UDP-sugar pyrophosphorylase	-3.61	0.0001
Manes.13G028800	-	enoyl-CoA hydratase/isomerase D	-3.62	0.00005
Manes.03G076800	-	Protein serine/threonine kinases;protein kinases;ATP binding;sugar binding;kinases;carbohydrate binding	-3.8	0.00005
Manes.02G087900	-	Predicted AT-hook DNA-binding family protein	-4.00	0.00005
Manes.08G019800	-	Programmed cell death 4	-4.07	0.00005
Manes.02G212800	GO:0043565,GO:0006355,GO:0003700,GO:0003677	Glutamine-dependent asparagine synthase 1	-4.2	0.0001
Manes.13G124700	-	Uncharacterized protein	-4.23	0.00005
Manes.01G242300	-	Homeobox 7	-4.25	0.0001
Manes.07G031300	GO:0055114,GO:0020037,GO:0016705,GO:0005506	HSP20-like chaperones superfamily protein	-4.28	0.0001
Manes.01G264600	GO:0008270,GO:0004089	Cytochrome P450, family 704, subfamily A, polypeptide 2	-4.47	0.00005
Manes.02G071200	GO:0045156,GO:0019684,GO:0009772	Cytochrome P450, family 77, subfamily A, polypeptide 5 pseudogene	-4.64	0.00005
Manes.17G116800	GO:0005524,GO:0006508,GO:0004222	Carbonic anhydrase 1	-4.65	0.00015
Manes.S113100	GO:0016798,GO:0016020	photosystem II reaction center protein A	-4.73	0.00005
Manes.02G046700	GO:0016984,GO:0015977,GO:0000287	FTSH protease 6	-4.83	0.00005
Manes.03G012600	GO:0016829,GO:0010333,GO:0000287	Glucuronidase 3	-4.91	0.00005
Manes.S113700	GO:0016829,GO:0010333,GO:0000287	Ribulose-bisphosphate carboxylases	-4.94	0.00005
Manes.02G086100	GO:0055114,GO:0020037,GO:00	Terpene synthase 21	-5.08	0.00005

	16705,GO:0005506			
Manes.08G138500,Manes.08G138600	GO:0016829,GO:0010333,GO:0008152,GO:0000287	Terpene synthase 21	-5.18	0.00005
Manes.01G109800	GO:0006468,GO:0005524,GO:0004672	Cytochrome P450, family 735, subfamily A, polypeptide 1	-5.20	0.00005
Manes.02G086300	-	Terpene synthase 21	-5.24	0.00005
Manes.02G172500	GO:0016829,GO:0010333,GO:0008152,GO:0000287	Protein kinase protein with adenine nucleotide alpha hydrolases-like domain	-5.30	0.00005
Manes.02G025400	GO:0016829,GO:0010333,GO:0008152,GO:0000287	Uncharacterised protein family (UPF0497)	-5.36	0.00005
Manes.12G076600	-	Terpene synthase 10	-5.38	0.00005
Manes.02G086200	GO:0055114,GO:0016491,GO:0006633,GO:0005506	Terpenoid cyclases/Protein prenyltransferases superfamily protein	-5.39	0.00015
Manes.02G221500	-	Protein of unknown function (DUF1442)	-5.42	0.0001
Manes.01G274900	-	Fatty acid hydroxylase superfamily	-5.46	0.00005
Manes.01G002600	GO:0055114,GO:0020037,GO:0006979,GO:0004601	Alpha/beta-Hydrolases superfamily protein	-5.52	0.00005
Manes.02G079400	-	Gamete expressed protein 1	-5.57	0.0001
Manes.02G026300	-	Peroxidase superfamily protein	-5.73	0.00005
Manes.01G095400	-	HXXXD-type acyl-transferase family protein	-5.81	0.00005
Manes.02G044700	GO:0033177,GO:0015991,GO:0015078	Expansin-like B1	-5.98	0.00005
Manes.02G067700	-	Regulator of Vps4 activity in the MVB pathway protein	-6.34	0.00005
Manes.S091000	-	ATP synthase subunit C family protein	-7.16	0.0001
Manes.03G027900	-	Oxidative stress 3	-7.27	0.00005
Manes.02G124700	-	HSP20-like chaperones superfamily protein	-7.45	0.00005
