# Supplementary material for: The early transcriptome response of cassava (Manihot esculenta Crantz) to mealybug (Phenacoccus manihoti) feeding
Source: PLoS One. 2018 Aug 22;13(8):e0202541. doi: 10.1371/journal.pone.0202541 (PMC6105004; doi:10.1371/journal.pone.0202541)
Supplement: S3 Table — (PDF) [file pone.0202541.s008.pdf]

S3 Table. List of P40/1 differentially expressed genes in cassava leaves in response to mealybug infestation at 24 and 72 hours post infestation normalized against mock (non-infested).

Gene ID	GO ID	Gene annotation	Log2(fold_change) 24 hpi	P-value
Manes.01G138100	GO:0005975,GO:0004553	Glycosyl hydrolase superfamily protein	5.53	0.00005
Manes.02G131900	-	Expansin A4	5.31	0.00005
Manes.03G048300	GO:0005515,GO:0006468,GO:0004672	Leucine-rich repeat transmembrane protein kinase family protein	4.92	0.00005
Manes.01G264600	GO:0055114,GO:0020037,GO:0016705,GO:0005506	Cytochrome P450, family 704, subfamily A, polypeptide 2	4.82	0.0001
Manes.03G043600	GO:0004857	Plant invertase/pectin methylesterase inhibitor superfamily protein	4.53	0.00005
Manes.01G212600	GO:0007165	Rho GTPase activating protein with PAK-box/P21-Rho-binding domain	4.38	0.00005
Manes.01G014700	GO:0008017,GO:0007018,GO:0005524,GO:0003777,GO:0005871	ATP binding microtubule motor family protein	4.30	0.00005
Manes.03G059300	GO:0006468,GO:0005524,GO:0004672,GO:0005515	Leucine-rich receptor-like protein kinase family protein	4.26	0.00005
Manes.05G012900	GO:0016746,GO:0008152	Glycerol-3-phosphate acyltransferase 3	3.88	0.00005
Manes.15G148400	-	Bifunctional inhibitor/lipid-transfer protein/seed storage 2S albumin superfamily protein	3.77	0.00005
Manes.01G189100	-	Cystathionine beta-synthase (CBS) protein	3.61	0.00015
Manes.15G130000	GO:0009607,GO:0006952	MLP-like protein 28	3.29	0.00005
Manes.02G174100	-	Nodulin MtN3 family protein	3.21	0.00005
Manes.02G104600	-	GAST1 protein homolog 1	3.11	0.0001
Manes.01G241000	GO:0016747,GO:0008610,GO:0016020,GO:0006633	3-ketoacyl-CoA synthase 1	2.85	0.00005
Manes.02G145900	GO:0006508,GO:0004190	Eukaryotic aspartyl protease family protein	2.80	0.00005
Manes.17G070900	GO:0003824	Camelliol C synthase 1	2.70	0.00015
Manes.05G143800	-	Expansin 11	2.17	0.00005
Manes.13G143700	GO:0046872	Zinc finger C-x8-C-x5-C-x3-H type family protein	2.16	0.00005
Manes.04G155800	GO:0008270,GO:0005515	RING/U-box superfamily protein	2.14	0.00005
Manes.07G059300	GO:0005975,GO:0004553	Glycosyl hydrolase superfamily protein	1.91	0.0002
Manes.12G031700	GO:0006355,GO:0003700	Ethylene response factor 1	1.62	0.00015
Manes.12G103400	GO:0046983	Basic helix-loop-helix (bHLH) DNA-binding superfamily protein	1.57	0.00005
Manes.10G105100	GO:0016887,GO:0005524,GO:0016020	White-brown complex homolog protein 11	1.55	0.00005
Manes.06G121800	-	hydroxysteroid dehydrogenase 6	1.43	0.0001
Manes.12G137800	GO:0008654,GO:0006021,GO:0004512	Myo-inositol-1-phosphate synthase 3	1.36	0.00005
Manes.02G017100	GO:0043565,GO:0006355,GO:0003700	WRKY DNA-binding protein 27	1.35	0.00005
Manes.02G189200	GO:0016788	GDSL-like Lipase/Acylhydrolase superfamily protein	1.30	0.00005
Manes.03G181100	-	probable membrane-associated kinase regulator 1	1.10	0.00005
Manes.06G121800	-	Hydroxysteroid dehydrogenase 6	1.07	0.0001
Manes.16G011800	-	Homeodomain-like superfamily protein	0.84	0.00005
Manes.01G018900	GO:0006468,GO:0004672	BAK1-interacting receptor-like kinase 1	-0.29	0.00005
Manes.05G167500	GO:0055114,GO:0020037,GO:0016705,GO:0005506	Cytochrome P450, family 82, subfamily C, polypeptide 2	-0.66	0.0002
Manes.02G203800	-	Hypothetical protein	-1.02	0.00005
Manes.02G040000	GO:0016021,GO:0016020,GO:0022857	Nodulin MtN21 /EamA-like transporter family protein	-1.12	0.00005
Manes.06G149700	-	Uncharacterized protein At3g49055	-1.14	0.00005
Manes.01G196500	GO:0016758,GO:0008152	UDP-glucosyl transferase 74B1	-1.2	0.0001
Manes.12G109400	-	RING/U-box superfamily protein	-1.61	0.00015
Manes.17G079800	GO:0009607,GO:0006952	MLP-like protein 28	-1.72	0.00005
Manes.06G156700	GO:0055085,GO:0022857,GO:0016021	Phosphate transporter 1;3	-1.88	0.00005

Manes.18G115900,Manes.18G116000	GO:0045017,GO:0004144	O-acyltransferase (WSD1-like) family protein	-2.19	0.00005
Manes.03G165000	-	Early nodulin-75-like	-2.31	0.0001
Manes.03G061200	-	Tetraspanin family protein	-2.32	0.00005
Manes.01G231100	GO:0055114,GO:0016491	Gibberellin 2-oxidase 6	-2.68	0.00005
Manes.08G154900	GO:0046983,GO:0008171	O-methyltransferase 1	-2.89	0.00005
Manes.10G105900	-	HSP20-like chaperones superfamily protein	-3.41	0.00005
Manes.02G099700	GO:0016787	Purple acid phosphatase 25	-3.49	0.00005
Manes.12G028900	GO:0003677	Basic helix-loop-helix (bHLH) DNA-binding superfamily protein	-3.63	0.00005
Manes.02G025100	-	Osmotin 34	-3.93	0.00035
Manes.07G040100	-	Hydroxyproline-rich glycoprotein family protein	-3.97	0.00015
Manes.01G212700	-	Uncharacterized protein	-4.36	0.00005
Manes.11G105200	GO:0016788	GDSL-like Lipase/Acylhydrolase superfamily protein	-4.52	0.00005
Manes.02G166800	GO:0005634	Cyclin A1;1	-4.56	0.00005
Manes.18G052600	-	SPX domain gene 2	-4.78	0.00005
Manes.01G274500	GO:0006629	Alpha/beta-Hydrolases superfamily protein	-5.15	0.00005
Manes.14G173500	GO:0016787	Purple acid phosphatase 17	-5.16	0.00005
Manes.02G002300	GO:0046983	Basic helix-loop-helix (bHLH) DNA-binding family protein	-5.23	0.00005
Manes.01G147400	-	Tetratricopeptide repeat (TPR)-like superfamily protein	-5.58	0.00025
Manes.02G039500	GO:0042545,GO:0030599,GO:0005618	Pectin lyase-like superfamily protein	-5.60	0.00015
Manes.02G164500	GO:0016787,GO:0008152	IAA-leucine resistant (ILR)-like gene 6	-5.65	0.00005
Manes.03G047200	GO:0016791,GO:0008152	Pyridoxal phosphate phosphatase-related protein	-5.66	0.00005
Manes.03G031600	-	Uncharacterized protein	-5.91	0.0001
Manes.02G189200	GO:0016788	GDSL-like Lipase/Acylhydrolase superfamily protein	-6.00	0.00005
Manes.S095100	-	Lipid transfer protein 3	-6.02	0.00005
Manes.01G148900	GO:0046872,GO:0030001	Heavy metal transport/detoxification superfamily protein	-6.16	0.0003
Manes.02G086200	GO:0016829,GO:0010333,GO:0008152,GO:0000287	Terpenoid cyclases/Protein prenyltransferases superfamily protein	-6.86	0.0004
Manes.S021300,Manes.S021400	GO:0031012,GO:0008270,GO:0006508,GO:0004222	Matrix metalloproteinase	-6.91	0.00005
Manes.02G124600	-	HSP20-like chaperones superfamily protein	-7.65	0.00005

**Gene ID**	**GO ID**	**Gene annotation**	**Log2(fold_change) 72 hpi**	
Manes.15G148400	-	Bifunctional inhibitor/lipid-transfer protein/seed storage 2S albumin superfamily protein	3.89	0.00005
Manes.01G087800	-	Expansin-like B1	3.73	0.00005
Manes.05G063900	GO:0016021	TSPO(outer membrane tryptophan-rich sensory protein)-related	3.72	0.00005
Manes.15G130200	GO:0009607,GO:0006952	Polyketide cyclase/dehydrase and lipid transport superfamily protein	3.68	0.00005
Manes.01G129500	GO:0005515,GO:0006468,GO:0005524,GO:0004672	Leucine-rich repeat protein kinase family protein	3.32	0.00005
Manes.18G101700,Manes.18G101800,Manes.18G101900	-	Terpenoid cyclases/Protein prenyltransferases superfamily protein	3.09	0.0001
Manes.13G143700	GO:0046872	Zinc finger C-x8-C-x5-C-x3-H type family protein	2.71	0.00005
Manes.02G115200	GO:0055085,GO:0016021	Auxin efflux carrier family protein	2.61	0.00005
Manes.11G137300	GO:0005975,GO:0004553	Chitinase	2.53	0.00005
Manes.05G143800		Expansin 11	2.42	0.00005
Manes.18G032200	GO:0055114,GO:0020037,GO:0016705,GO:0005506	Cytochrome P450, family 82, subfamily C, polypeptide 4	2.30	0.00005
Manes.12G103400	GO:0046983	Basic helix-loop-helix (bHLH) DNA-binding superfamily protein	2.14	0.00005
Manes.02G039500	GO:0042545,GO:0030599,GO:0005618	Pectin lyase-like superfamily protein	2.09	0.00005
Manes.02G151200	-	Phosphate-responsive 1 family protein	2.05	0.00015
Manes.01G196200	GO:0016787,GO:0008152	IAA-leucine resistant (ILR)-like gene	1.88	0.00005

		2		
Manes.13G101600	GO:0008152,GO:0003824,GO:0016114,GO:0008661	Deoxyxylulose-5-phosphate synthase	1.85	0.00005
Manes.06G030200	-	Ribonuclease H-like superfamily protein	1.78	0.00005
Manes.13G056600	GO:0016758,GO:0008152	UDP-Glycosyltransferase superfamily protein	1.77	0.0001
Manes.13G141100	GO:0016021,GO:0006952	Seven transmembrane MLO family protein	1.70	0.00005
Manes.S043400,Manes.S043500	GO:0005975,GO:0004553	Beta glucosidase 40	1.47	0.00005
Manes.04G159500	GO:0055085,GO:0016021,GO:0016020,GO:0006810,GO:0005215	Major facilitator superfamily protein	1.28	0.00015
Manes.05G029900	GO:0030170,GO:0009058	ACC synthase 1	1.25	0.00005
Manes.01G189000	GO:0043565,GO:0006355,GO:0003700	WRKY DNA-binding protein 72	1.03	0.00005
Manes.07G077900	GO:0055114,GO:0020037,GO:0016705,GO:0005506	Cytochrome P450, family 707, subfamily A, polypeptide 4	0.92	0.00005
Manes.12G076900	GO:0016829,GO:0010333,GO:0008152,GO:0000287	Terpene synthase 02	0.59	0.00005
Manes.06G108300		Raffinose synthase family protein	0.09	0.0001
Manes.02G040000	GO:0016021,GO:0016020,GO:0022857	Nodulin MtN21 /EamA-like transporter family protein	-0.69	0.00005
Manes.17G017700	-	Tetratricopeptide repeat (TPR)-like superfamily protein	-0.76	0.00005
Manes.12G109400	-	RING/U-box superfamily protein	-1.22	0.0004
Manes.02G105300	-	Pathogenesis-related thaumatin superfamily protein	-1.52	0.00005
Manes.18G040300	GO:0003677	WUSCHEL related homeobox 4	-1.57	0.00005
Manes.06G026500	GO:0048544,GO:0006468,GO:0004672	S-locus lectin protein kinase family protein	-1.63	0.00005
Manes.06G066800	GO:0006308,GO:0004519,GO:0003676	Endonuclease 2	-1.71	0.00005
Manes.14G118700	GO:0055114,GO:0016491	2-oxoglutarate (2OG) and Fe(II)-dependent oxygenase superfamily protein	-1.79	0.00015
Manes.01G220700	-	Protein of unknown function, DUF538	-1.81	0.00015
Manes.02G065400	GO:0055114,GO:0016616,GO:0006694,GO:0003854	NAD(P)-binding Rossmann-fold superfamily protein	-1.96	0.0001
Manes.01G196500	GO:0016758,GO:0008152	UDP-glucosyl transferase 74B1	-2.23	0.0002
Manes.03G061200		Tetraspanin family protein	-2.54	0.00015
Manes.08G154900	GO:0046983,GO:0008171	O-methyltransferase 1	-2.56	0.00005
Manes.01G041100	GO:0005515	Disease resistance family protein / LRR family protein	-2.85	0.0002
Manes.16G039100	-	Hypothetical protein	-2.85	0.00005
Manes.17G075600	-	Myb domain protein 4	-2.95	0.00015
Manes.01G204500	GO:0047746,GO:0015996	Chlorophyllase 1	-3.02	0.0001
Manes.07G064200	GO:0005525	ADP-ribosylation factor family protein	-3.12	0.00005
Manes.07G050700	-	CAP (Cysteine-rich secretory proteins, Antigen 5, and Pathogenesis-related 1 protein) superfamily protein	-3.26	0.0001
Manes.02G164500	GO:0016787,GO:0008152	IAA-leucine resistant (ILR)-like gene 6	-3.31	0.0001
Manes.13G127800	GO:0055085,GO:0016020,GO:0015297,GO:0015238,GO:0006855	MATE efflux family protein	-3.35	0.0002
Manes.02G099700	GO:0016787	Purple acid phosphatase 25	-3.4	0.0002
Manes.01G250800	GO:0051087	BCL-2-associated athanogene 6	-3.46	0.00015
Manes.02G205700	GO:0007165,GO:0005515,GO:0043531	Disease resistance protein (TIR-NBS-LRR class), putative	-3.57	0.00005
Manes.02G035800	GO:0055114,GO:0016491,GO:0005507	Cupredoxin superfamily protein	-3.88	0.0002
Manes.02G086100,Manes.02G086200,Manes.02G086300	GO:0016829,GO:0010333,GO:0000287	Terpene synthase 21	-3.98	0.00005
Manes.07G070800	GO:0016758,GO:0008152	UDP-Glycosyltransferase superfamily protein	-4.02	0.0001
Manes.01G241000	GO:0016747,GO:0008610,GO:0016020,GO:0006633	3-ketoacyl-CoA synthase 1	-4.07	0.00015
Manes.11G105200	GO:0016788	GDSL-like Lipase/Acylhydrolase superfamily protein	-4.15	0.0001
Manes.12G028900	GO:0003677	Basic helix-loop-helix (bHLH) DNA-binding superfamily protein	-4.26	0.00005
Manes.02G025100	-	Osmotin 34	-4.31	0.00005

Manes.02G086300	GO:0016829,GO:0010333,GO:0008152,GO:0000287	Terpene synthase 21	-4.52	0.0001
Manes.02G028300	-	Osmotin 34	-4.86	0.00035
Manes.13G125100	GO:0006355,GO:0003700	Integrase-type DNA-binding superfamily protein	-5.10	0.00005
Manes.01G203500	-	Calmodulin-like 11	-5.11	0.00005
Manes.01G274500	GO:0006629	Alpha/beta-Hydrolases superfamily protein	-5.17	0.00005
Manes.S021200	GO:0031012,GO:0008270,GO:0006508,GO:0004222	Matrixin family protein	-5.35	0.0001
Manes.01G076500	GO:0004575,GO:0004564	Glycosyl hydrolases family 32 protein	-5.58	0.00005
Manes.14G173500	GO:0016787	Purple acid phosphatase 17	-5.81	0.00005
Manes.02G189200	GO:0016788	GDSL-like Lipase/Acylhydrolase superfamily protein	-6.01	0.00005
Manes.01G142100	GO:0005515	Leucine-rich repeat (LRR) family protein	-6.16	0.0001
Manes.04G089800	GO:0008081,GO:0006629	Senescence-related gene 3	-6.17	0.00005
Manes.08G055600	-	Hypothetical protein	-6.17	0.00005
Manes.02G124700	-	HSP20-like chaperones superfamily protein	-7.07	0.0003

**Gene ID**	**GO ID**	**Gene annotation**	**Log2(fold_change) 24 hpi vs 72 hpi**	
Manes.02G151000	-	Phosphate-responsive 1 family protein	5.48	0.00005
Manes.04G029500	GO:0055114,GO:0016491	Exocyst subunit exo70 family protein H7	5.42	0.00005
Manes.05G195300	GO:0016021,GO:0015012,GO:0006024	Transmembrane amino acid transporter family protein	5.38	0.0001
Manes.03G044100	GO:0043565,GO:0006355,GO:0005634,GO:0003700	Integrase-type DNA-binding superfamily protein	5.31	0.00005
Manes.01G121300	GO:0016758,GO:0008152	ARM repeat superfamily protein	5.13	0.0004
Manes.07G072600	GO:0050832,GO:0042742	Matrixin family protein	5.04	0.00005
Manes.13G125100	-	Integrase-type DNA-binding superfamily protein	4.98	0.00055
Manes.S055900	-	Xyloglucan endotransglycosylase 6	4.64	0.00005
Manes.S002300	GO:0016829,GO:0010333,GO:0008152,GO:0000287	C2H2 and C2HC zinc fingers superfamily protein	4.63	0.00005
Manes.02G124700,Manes.02G124800	-	HSP20-like chaperones superfamily protein	4.51	0.00005
Manes.18G136400	-	Protein phosphatase 2C family protein	4.19	0.00005
Manes.05G113200	-	Calmodulin like 37	4.15	0.0005
Manes.02G146000	-	Dicarboxylate carrier 2	3.98	0.00035
Manes.02G130000	-	Zinc finger (C3HC4-type RING finger) family protein	3.69	0.00005
Manes.06G020900	GO:0016747,GO:0008610,GO:0016020,GO:0006633	Glycosyltransferase family protein 47	3.58	0.0001
Manes.16G116900	-	Arabinogalactan protein 22	3.55	0.00005
Manes.18G034400	-	Protein of unknown function (DUF1645)	3.52	0.0001
Manes.11G135900	GO:0043531	Exocyst subunit exo70 family protein H7	3.35	0.00005
Manes.02G017100	-	WRKY DNA-binding protein 27	3.32	0.00005
Manes.03G118300	GO:0055114,GO:0020037,GO:0016705,GO:0005506	Calcium-binding EF-hand family protein	3.27	0.00005
Manes.09G046000	-	plant U-box 23	3.26	0.00005
Manes.02G125200	GO:0003824,GO:0006470,GO:0004722	HSP20-like chaperones superfamily protein	3.17	0.00005
Manes.17G071600	-	Jasmonate-zim-domain protein 8	3.10	0.00015
Manes.18G065800	GO:0006355,GO:0003700	Phosphate-responsive 1 family protein	2.85	0.00005
Manes.02G110100	-	RING/U-box superfamily protein	2.84	0.0003
Manes.09G112700	-	WRKY DNA-binding protein 33	2.82	0.00005
Manes.03G106300	-	Ethylene responsive element binding factor 4	2.47	0.00005
Manes.05G030900	GO:0005515	WRKY family transcription factor	2.37	0.00005
Manes.05G165900	-	Uncharacterised protein family (UPF0114)	2.36	0.00005
Manes.07G063300	GO:0031012,GO:0008270,GO:0006508,GO:0004222	Protein phosphatase 2C family protein	2.33	0.00015
Manes.15G189500	-	HSP20-like chaperones superfamily protein	1.78	0.00005
Manes.05G038800	-	BON association protein 2	1.73	0.00005
Manes.13G141100	-	Seven transmembrane MLO family protein	1.49	0.00005

Manes.09G113500	GO:0006887,GO:0000145	Hypothetical protein	1.45	0.00005
Manes.18G118900	GO:0003824,GO:0006470,GO:0004722	Integrase-type DNA-binding superfamily protein	1.35	0.00005
Manes.06G051100	-	3-ketoacyl-CoA synthase 11	0.82	0.0003
Manes.04G005600	GO:0006887,GO:0000145	Cytochrome P450, family 94, subfamily C, polypeptide 1	0.54	0.00005
Manes.13G152300	-	Wound-responsive family protein	0.23	0.00025
Manes.S051300	GO:0048046,GO:0016762,GO:0006073,GO:0005618,GO:0005975,GO:0004553	Terpenoid cyclases/Protein prenyltransferases superfamily protein	-0.32	0.00005
Manes.13G056600	GO:0043565,GO:0006355,GO:0005634,GO:0003700	UDP-Glycosyltransferase superfamily protein	-0.59	0.00005
Manes.04G109700	-	2-oxoglutarate (2OG) and Fe(II)-dependent oxygenase superfamily protein	-0.75	0.00035
Manes.14G143500	GO:0043531	Heat shock protein 70B	-0.96	0.00005
Manes.03G040200	GO:0006355,GO:0003700	Acyl-CoA N-acyltransferases (NAT) superfamily protein	-0.99	0.00005
Manes.S067700	-	Tetratricopeptide repeat (TPR)-like superfamily protein	-1.03	0.00005
Manes.08G135300	GO:0016567,GO:0004842	Pathogenesis-related 4	-1.09	0.00025
Manes.11G156500	-	Disease resistance protein (TIR-NBS-LRR class) family	-1.11	0.00005
Manes.12G024900	-	Alpha/beta-Hydrolases superfamily protein	-1.13	0.00005
Manes.09G051500	GO:0043565,GO:0006355,GO:0003700	TRICHOME BIREFRINGENCE-LIKE 8	-1.29	0.00025
Manes.16G025700	-	Aluminium induced protein with YGL and LRDR motifs	-1.36	0.00005
Manes.02G044700	GO:0016829,GO:0010333,GO:0000287	Expansin-like B1	-1.40	0.00025
Manes.18G023000	GO:0055114,GO:0020037,GO:0016705,GO:0005506	uncharacterized protein LOC110606054	-1.42	0.00005
Manes.14G165100	-	Disease resistance protein (TIR-NBS-LRR class) family	-1.45	0.00005
Manes.13G139000	GO:0016021,GO:0006952	Late embryogenesis abundant domain-containing protein / LEA domain-containing protein	-1.47	0.00005
Manes.02G221500	GO:0008080	Protein of unknown function (DUF1442)	-1.61	0.00005
Manes.04G156300	GO:0043565,GO:0006355,GO:0003700	Protein LNK3-like	-1.68	0.00005
Manes.18G032200	-	Cytochrome P450, family 82, subfamily C, polypeptide 4	-2.04	0.00015
Manes.02G128000	GO:0008270,GO:0005515	Highly ABA-induced PP2C gene 3	-2.47	0.00005
Manes.01G119000	GO:0016567,GO:0004842	HSP20-like chaperones superfamily protein	-3.02	0.00005
Manes.01G117900	-	Senescence-associated family protein	-3.12	0.00015
Manes.02G086100,Manes.02G086200,Manes.02G086300	GO:0008270,GO:0005515	Terpene synthase 21	-3.61	0.0002
Manes.13G124500	GO:0006355,GO:0003700	Heat shock factor 4	-3.73	0.00005
Manes.06G091300	GO:0051082,GO:0006950,GO:0006457,GO:0005524	Expansin A1	-3.89	0.00005
Manes.01G196500	GO:0043565,GO:0006355,GO:0003700	UDP-glucosyl transferase 74B1	-4.21	0.00015
Manes.08G135200	GO:0008061,GO:0050832,GO:0042742	Pathogenesis-related 4	-4.39	0.0001
Manes.02G124600	-	HSP20-like chaperones superfamily protein	-4.71	0.00005
Manes.01G116700	-	Calmodulin like 23	-4.71	0.0001
Manes.03G049500	GO:0006355,GO:0003700	Heat shock transcription factor A2	-4.91	0.0002
Manes.06G152800	GO:0003824,GO:0006470,GO:0004722	Heat shock protein 90.1	-5.21	0.00005
Manes.13G013400	GO:0016758,GO:0008152	Heat shock protein 21	-5.31	0.00005
