# Supplementary material for: The early transcriptome response of cassava (Manihot esculenta Crantz) to mealybug (Phenacoccus manihoti) feeding
Source: PLoS One. 2018 Aug 22;13(8):e0202541. doi: 10.1371/journal.pone.0202541 (PMC6105004; doi:10.1371/journal.pone.0202541)
Supplement: S4 Table — (PDF) [file pone.0202541.s009.pdf]

Table S4: Gene Ontologies (GO) for AR23.1 and P40/1 cassava genotypes following mealybug infestation categorized in Biological process, Molecular function and Cellular component.

GO term	Gene ID	GO ID	Gene annotation
**Biological process**			
**1. Metabolic process**	Manes.02G054300	GO:0016758,GO:0008152	UDP-Glycosyltransferase superfamily protein
	Manes.02G071200	GO:0008152,GO:0003824	cytochrome P450, family 77, subfamily A, polypeptide 5 pseudogene
	Manes.02G086200	GO:0016829,GO:0010333,GO:0008152,GO:0000287	Terpenoid cyclases/Protein prenyltransferases superfamily protein
	Manes.12G076600	GO:0016829,GO:0010333,GO:0008152,GO:0000287	Terpene synthase 10
	Manes.16G016100	GO:0016829,GO:0010333,GO:0008152,GO:0000287	Terpene synthase 14
	Manes.S099100	GO:0016829,GO:0010333,GO:0008152,GO:0000287	Terpene synthase-like sequence-1,8-cineole
	Manes.01G231100	GO:0055114,GO:0016491	Tibberellin 2-oxidase 6
	Manes.01G274900	GO:0055114,GO:0016491,GO:0006633,GO:0005506	Fatty acid hydroxylase superfamily
	Manes.03G032500	GO:0055114,GO:0016491	ACC oxidase 1
	Manes.04G120100	GO:0055114,GO:0016491	2-oxoglutarate (2OG) and Fe(II)-dependent oxygenase superfamily protein
	Manes.08G002700	GO:0055114,GO:0020037,GO:0006979,GO:0004601	Tscorbate peroxidase 2
	Manes.10G071700	GO:0055114,GO:0016491,GO:0051536,GO:0020037	Titrite reductase 1
	Manes.10G146700	GO:0055114,GO:0020037,GO:0016705,GO:0005506	Tytochrome P450, family 705, subfamily A, polypeptide 27
	Manes.14G051800	GO:0055114,GO:0020037,GO:0016705,GO:0005506	Tytochrome P450, family 94, subfamily B, polypeptide 3
	Manes.15G121600	GO:0055114,GO:0016491	Tibberellin 2-oxidase
	Manes.S113100	GO:0045156,GO:0019684,GO:0009772	Thotosystem II reaction center protein A
	Manes.01G110800	GO:0008270,GO:0005975,GO:0004476,GO:0009298	Mannose-6-phosphate isomerase, type I
	Manes.S113700	GO:0016984,GO:0015977,GO:0000287	Tibulose-bisphosphate carboxylases
	Manes.01G192100	GO:0045454,GO:0015035,GO:0006662	Thioredoxin H-type 7
	Manes.02G212800	GO:0006529,GO:0004066	Glutamine-dependent asparagine synthase 1

	Manes.01G242300	GO:0043565,GO:0006355,GO:0003700,GO:0003677	Homeobox 7
	Manes.03G044100	GO:0006355,GO:0003700	Integrase-type DNA-binding superfamily protein
	Manes.06G131100	GO:0006355,GO:0003700	Ethylene responsive element binding factor 1
	Manes.13G112500	GO:0008270,GO:0006355,GO:0005634,GO:0004402,GO:0003712,GO:0005515	BTB and TAZ domain protein 4
	Manes.03G120700	GO:0045454,GO:0005681,GO:0000398	mRNA splicing factor, thioredoxin-like U5 snRNP
**2. Cellular process**	Manes.01G067400	GO:0006355,GO:0003700	C-repeat-binding factor 4
	Manes.01G085400	GO:0006355,GO:0003700	Ethylene responsive element binding factor 1
	Manes.02G089000	GO:0006355,GO:0003677	NAC domain containing protein 42
	Manes.03G056100	GO:0006355,GO:0003700	Integrase-type DNA-binding superfamily protein
	Manes.06G131100	GO:0006355,GO:0003700	Ethylene responsive element binding factor 1
	Manes.13G112500	GO:0008270,GO:0006355,GO:0005634,GO:0004402,GO:0003712,GO:0005515	BTB and TAZ domain protein 4
	Manes.03G044100	GO:0006355,GO:0003700	Integrase-type DNA-binding superfamily protein
	Manes.15G015600	GO:0045454,GO:0015035,GO:0009055	Glutaredoxin family protein
	Manes.15G094100	GO:0048544	D-mannose binding lectin protein with Apple-like carbohydrate-binding domain
	Manes.07G086000	GO:0020037,GO:0017004	Cytochrome C assembly protein
	Manes.05G132500	GO:0008017,GO:0007018,GO:0005524,GO:0003777,GO:0005871	ATP binding microtubule motor family protein
**3. Response to stress**	Manes.05G061300	GO:0006950	Late embryogenesis abundant 3 (LEA3) family protein
	Manes.01G065500	GO:0055114,GO:0020037,GO:0006979,GO:0004601	Peroxidase superfamily protein
	Manes.02G025800	GO:0055114,GO:0020037,GO:0006979,GO:0004601	Peroxidase superfamily protein
	Manes.02G026300	GO:0055114,GO:0020037,GO:0006979,GO:0004601	Peroxidase superfamily protein
	Manes.08G002700	GO:0055114,GO:0020037,GO:0006979,GO:0004601	Escorbate peroxidase 2
	Manes.02G015400	GO:0009378,GO:0006310,GO	P-loop containing nucleoside

		:0006281	triphosphate hydrolases superfamily protein
	Manes.01G020600	GO:0030983,GO:0006298,GO:0005524	MUTS homolog 2
	Manes.01G082400	GO:0030983,GO:0006298,GO:0005524	MUTS-like protein 4
	Manes.02G106500	GO:0016021,GO:0006952	Seven transmembrane MLO family protein
	Manes.15G130200	GO:0009607,GO:0006952	Polyketide cyclase/dehydrase and lipid transport superfamily protein
	Manes.15G130300	GO:0009607,GO:0006952	MLP-like protein 43
	Manes.13G141100	GO:0016021,GO:0006952	Seven transmembrane MLO family protein
	Manes.15G130000	GO:0009607,GO:0006952	MLP-like protein 28
	Manes.17G079800	GO:0009607,GO:0006952	MLP-like protein 28
	Manes.08G135200	GO:0050832,GO:0042742	Pathogenesis-related 4
	Manes.08G135300	GO:0008061,GO:0050832,GO:0042742	Pathogenesis-related 4
**Molecular function**			
**1. Catalytic activity**	Manes.02G071200	GO:0008152,GO:0003824	Pytochrome P450, family 77, subfamily A, polypeptide 5 pseudogene
	Manes.02G128000	GO:0003824,GO:0006470,GO:0004722	Highly ABA-induced PP2C gene 3
	Manes.17G070900	GO:0003824	Camelliol C synthase 1
	Manes.08G138500	GO:0016829,GO:0010333,GO:0000287	Terpene synthase 21
	Manes.12G076600	GO:0016829,GO:0010333,GO:0008152,GO:0000287	Terpene synthase 10
	Manes.16G016100	GO:0016829,GO:0010333,GO:0008152,GO:0000287	Terpene synthase 14
	Manes.S113700	GO:0016984,GO:0015977,GO:0000287	Ribulose-bisphosphate carboxylases
	Manes.17G116800	GO:0008270,GO:0004089	Carbonic anhydrase 1
	Manes.S099100	GO:0016829,GO:0010333,GO:0008152,GO:0000287	terpene synthase-like sequence-1,8-cineole
	Manes.03G018600	GO:0016887,GO:0005524,GO:0016020	Pleiotropic drug resistance 12
	Manes.02G039000	GO:0006508,GO:0004190	Eukaryotic aspartyl protease family protein
	Manes.02G145900	GO:0006508,GO:0004190	Eukaryotic aspartyl protease family protein
	Manes.16G132600	GO:0006508,GO:0004190	Eukaryotic aspartyl protease family protein
	Manes.07G059300	GO:0005975,GO:0004553	Glycosyl hydrolase superfamily protein
	Manes.01G076500	GO:0004575,GO:0004564	Glycosyl hydrolases family 32 protein

**2. Binding**	Manes.01G231200	GO:0042545,GO:0030599,GO:0005618	Pectin lyase-like superfamily protein
	Manes.01G110800	GO:0008270,GO:0005975,GO:0004476,GO:0009298	Mannose-6-phosphate isomerase, type I
	Manes.13G112500	GO:0008270,GO:0006355,GO:0005634,GO:0004402,GO:0003712,GO:0005515	BTB and TAZ domain protein 4
	Manes.01G013000	GO:0006468,GO:0005524,GO:0004672	Wall-associated kinase family protein
	Manes.01G020600	GO:0030983,GO:0006298,GO:0005524	MUTS homolog 2
	Manes.01G070800	GO:0006355,GO:0005634,GO:0005524,GO:0032502,GO:0006351	Growth-regulating factor 2
	Manes.02G170200	GO:0005509,GO:0006468,GO:0005524,GO:0004672	Wall associated kinase 3
	Manes.02G172500	GO:0006468,GO:0005524,GO:0004672	Protein kinase protein with adenine nucleotide alpha hydrolases-like domain
	Manes.03G018600	GO:0016887,GO:0005524,GO:0016020	Pleiotropic drug resistance 12
	Manes.03G076800	GO:0006468,GO:0005524,GO:0004672	protein serine/threonine kinases;protein Kinases;ATP binding;sugar binding;kinases;carbohydrate binding
	Manes.05G132500	GO:0008017,GO:0007018,GO:0005524,GO:0003777,GO:0005871	ATP binding microtubule motor family protein
	Manes.06G154000	GO:0016887,GO:0005524,GO:0016020	ABC-2 type transporter family protein
	Manes.10G105100	GO:0016887,GO:0005524,GO:0016020	White-brown complex homolog protein 11
	Manes.03G056100	GO:0006355,GO:0003700	Integrase-type DNA-binding superfamily protein
	Manes.06G131100	GO:0006355,GO:0003700	Ethylene responsive element binding factor 1
	Manes.08G138500	GO:0016829,GO:0010333,GO:0000287	Terpene synthase 21
	Manes.12G076600	GO:0016829,GO:0010333,GO:0008152,GO:0000287	Terpene synthase 10
	Manes.16G016100	GO:0016829,GO:0010333,GO:0008152,GO:0000287	Terpene synthase 14
	Manes.S099100	GO:0016829,GO:0010333,GO:0008152,GO:0000287	Terpene synthase-like sequence-1,8-cineole
	Manes.S113700	GO:0016984,GO:0015977,GO	Ribulose-bisphosphate carboxylases

		:0000287	
	Manes.10G146700	GO:0055114,GO:0020037,GO:0016705,GO:0005506	Cytochrome P450, family 705, subfamily A, polypeptide 27
	Manes.14G051800	GO:0055114,GO:0020037,GO:0016705,GO:0005506	Cytochrome P450, family 94, subfamily B, polypeptide 3
	Manes.08G002700	GO:0055114,GO:0020037,GO:0006979,GO:0004601	Ascorbate peroxidase 2
	Manes.17G098200	GO:0020037,GO:0019825	Hemoglobin 1
**Cellular component**			
**1. Cell**	Manes.13G112500	GO:0008270,GO:0006355,GO:0005634,GO:0004402,GO:0003712,GO:0005515	BTB and TAZ domain protein 4
	Manes.14G006300	GO:0005515	CCT motif family protein
	Manes.14G096200	GO:0005515	RNI-like superfamily protein
	Manes.03G066400	GO:0016021,GO:0015116,GO:0008272,GO:0055085,GO:0016020,GO:0008271,GO:1902358	Sulfate transporter 3;5
	Manes.04G094700	GO:0071805,GO:0016020,GO:0015079	K+ uptake transporter 3
	Manes.06G154000	GO:0016887,GO:0005524,GO:0016020	ABC-2 type transporter family protein
	Manes.09G062300	GO:0016020,GO:0006810,GO:0005215	Gamma tonoplast intrinsic protein
	Manes.10G105100	GO:0016887,GO:0005524,GO:0016020	white-brown complex homolog protein 11
	Manes.01G086900	GO:0055085,GO:0016021	Major facilitator superfamily protein
	Manes.01G161800	GO:0055085,GO:0016021	Cation exchanger 3
	Manes.02G040000	GO:0016021,GO:0016020,GO:0022857	Nodulin MtN21 /EamA-like transporter family protein
	Manes.02G106500	GO:0016021,GO:0006952	Seven transmembrane MLO family protein
	Manes.13G112500	GO:0008270,GO:0006355,GO:0005634,GO:0004402,GO:0003712,GO:0005515	BTB and TAZ domain protein 4
**2. Cell part**	Manes.05G132500	GO:0008017,GO:0007018,GO:0005524,GO:0003777,GO:0005871	ATP binding microtubule motor family protein
	Manes.03G120700	GO:0045454,GO:0005681,GO:0000398	mRNA splicing factor, thioredoxin-like U5 snRNP
	Manes.02G039500	GO:0042545,GO:0030599,GO:0005618	Pectin lyase-like superfamily protein
	Manes.01G067000	GO:0055085,GO:0016020,GO:0015297,GO:0015238,GO:0006855	MATE efflux family protein

	Manes.01G108600	GO:0016020	Uncharacterized protein family (UPF0016)
	Manes.01G254100	GO:0016757,GO:0016020	Protein of unknown function (DUF604)
	Manes.02G032300	GO:0055085,GO:0016020,GO:0015297,GO:0015238,GO:0006855	MATE efflux family protein
	Manes.02G040000	GO:0016021,GO:0016020,GO:0022857	Nodulin MtN21 /EamA-like transporter family protein
	Manes.02G051000	GO:0055085,GO:0016020,GO:0006811,GO:0005216	Cyclic nucleotide-regulated ion channel family protein
	Manes.S091000	GO:0033177,GO:0015991,GO:0015078	ATP synthase subunit C family protein
	Manes.02G051100	GO:0055085,GO:0016020,GO:0006811,GO:0005216	Cyclic nucleotide-regulated ion channel family protein
	Manes.13G112500	GO:0008270,GO:0006355,GO:0005634,GO:0004402,GO:0003712,GO:0005515	BTB and TAZ domain protein 4
	Manes.03G120700	GO:0045454,GO:0005681,GO:0000398	mRNA splicing factor, thioredoxin-like U5 snRNP
	Manes.05G132500	GO:0008017,GO:0007018,GO:0005524,GO:0003777,GO:0005871	ATP binding microtubule motor family protein
	Manes.02G039500	GO:0042545,GO:0030599,GO:0005618	Pectin lyase-like superfamily protein
